# SlKNUCKLES regulates floral meristem activity and controls fruit size in *Solanum lycopersicum*

**DOI:** 10.1093/hr/uhae331

**Published:** 2024-11-21

**Authors:** Dongbao Li, Wen Yang, Zhiyue Wu, Yonghua Yang, Zhongling Wen, Bo Sun

**Affiliations:** State Key Laboratory of Pharmaceutical Biotechnology, School of Life Sciences, Nanjing University, Nanjing 210023, China; State Key Laboratory of Pharmaceutical Biotechnology, School of Life Sciences, Nanjing University, Nanjing 210023, China; State Key Laboratory of Pharmaceutical Biotechnology, School of Life Sciences, Nanjing University, Nanjing 210023, China; State Key Laboratory of Pharmaceutical Biotechnology, School of Life Sciences, Nanjing University, Nanjing 210023, China; State Key Laboratory of Pharmaceutical Biotechnology, School of Life Sciences, Nanjing University, Nanjing 210023, China; State Key Laboratory of Pharmaceutical Biotechnology, School of Life Sciences, Nanjing University, Nanjing 210023, China; Jiangsu Collaborative Innovation Center of Regional Modern Agriculture & Environment Protection, Huaiyin Normal University, Huai'an 223300, China

## Abstract

Timed termination of floral meristem (FM) is crucial for proper development of floral organs and fruits. In *Solanum lycopersicum*, *CLAVATA3* (*CLV3*)-*WUSCHEL* (*WUS*) feedback regulation maintains FM homeostasis in early stage of floral buds. It is known that the zinc finger protein SlKNUCKLES (SlKNU) functions to promote FM determinacy by directly repressing the stem cell identity gene *SlWUS*. However, how the robust FM activity is suppressed to secure fruit development is not fully understood in tomato. Here, we demonstrate that SlKNU also directly represses the stem cell marker gene *SlCLV3* and the receptor gene *SlCLV1* for FM determinacy control. Besides, loss-of-function mutants of *SlKNU* generated by CRISPR-Cas9 show increased fruit size of tomato. Moreover, overexpression of *SlKNU* attenuates the activities of the shoot apical meristem (SAM) and FM in *Arabidopsis*, but normal carpel development is still maintained. Hence, although the function of KNU in tomato and *Arabidopsis* may diverge during evolution, the role of KNU for FM determinacy and fruit size control is conserved and may potentially be useful for enhancing fruit yield of tomato.

## Introduction

Within the shoot apical meristem (SAM) of plants, there exists a group of slowly dividing pluripotent cells with the potential to differentiate into various plant cell types, which are crucial for the upward growth of the plant [1, 2]. Tomato is a typical sympodial plant capable of multiple floral transitions, unlike *Arabidopsis*, which undergo only a single floral transition [3]. In controlled or indoor environments, the Micro-Tom tomato (*Solanum lycopersicum* Sl) usually exhibits three to four floral transitions. Each transition leads to the development of a new inflorescence and subsequent fruit [4, 5]. When tomato shifts from vegetative to reproductive growth, the SAM undergoes a transformation into a transitional meristem (TM). This TM then differentiates into an inflorescence meristem (IM) and a floral meristem (FM) [6]. The FM subsequently differentiates further to give rise to various floral organs, including sepals, petals, stamens, and carpels [7]. Fruit development originates from the fusion of carpels, a highly specialized organ. In *Arabidopsis*, the ovary formed by the fusion of two carpels eventually develops into fruit [8]. Similarly, the development of fruit in tomato also arises from the fusion of carpels [9]. Therefore, precise regulation of FM is not only crucial for the formation of flowers but also forms the basis of fruit development and plays a significant role in plant reproduction.

In *Arabidopsis*, the *CLAVATA3 (CLV3)-WUSCHEL (WUS)* feedback regulation plays a crucial role in maintaining the activity of the FM [10]. *WUS*, the stem cell identity gene, is essential for the maintenance of stem cells [11]. Expression of *WUS* is confined to the organizing center (OC), and the WUS protein can migrate upwards through plasmodesmata to the central zone (CZ) and activate the expression of *CLV3* by binding to its promoter [12]. As the marker gene for stem cell activity, *CLV3* encodes a small peptide, which acts as a signaling molecule and diffuses to the quiescent center (QC), where CLV3 can be perceived by transmembrane receptor complexes formed by CLV1, CLV2, CORYNE (CRN), BARELY ANY MERISTEMS (BAMs), RECEPTOR-LIKE PROTEIN KINASE 2 (RPK2), or CLAVATA3-INSENSITIVE RECEPTOR KINASES (CIKs) on the membrane [13, 14]. Through a cascade of signaling reactions, CLV3 inhibits the expression of *WUS*.

CLV3 is a member of the CLV3/EMBRYO-SURROUNDING REGION (CLE) peptide family [15]. In tomato, the absence of *SlCLV3* leads to a significant increase in the transcription level of its close homolog *SlCLE9*, which may actively compensate for the loss of *SlCLV3* [16]. The *CLV3-WUS* feedback regulatory pathway, which maintains the size of the stem cell population, is highly conserved among different plant species [17, 18]. Wild-type (WT) tomato fruits typically have two to three locules, representing the most primitive state. However, a quantitative trait locus (QTL) allele known as locule number (*lc*) has been identified in tomato, whose mutation leads to a gain-of-function mutation in the *SlWUS*, which significantly increases the number of locules within the tomato fruit [19]. Another QTL allele, *fasciated* (*fas*), results in reduced activity of the *SlCLV3* promoter, thereby also producing more locules [19, 20]. Strikingly, the homozygous double mutants of these two alleles exhibit a drastic increase in the number of locules [19]. Additionally, *SlWUS* RNA interference materials showed tomato flowers with significantly decreased size, and notably smaller fruit size compared to WT. The number of locules in *SlWUS-RNAi* tomato fruits is also obviously reduced [21]. Disruption of the *SlWUS*-*SlCLV3* regulatory loop leads to the destabilization of FM, directly causing abnormal flower development and resulting in malformed fruits. It has been shown that the increase of FM size and stem cell numbers can significantly enhance maize and tomato yield [16, 22].

In both *Arabidopsis* and tomato, the *CLV3-WUS* feedback regulation plays an indispensable role in FM maintenance. In *Arabidopsis*, KNUCKLES (AtKNU), a C2H2 zinc finger protein, can directly bind to the promoter regions of *WUS* and *CLV3* to suppress their expression and thereby timely terminates FM activity to ensure proper carpel development [14, 23]. In tomato, an AtKNU homolog protein named SlKNUCKLES (SlKNU) is reported to be involved in a transcriptional repression complex formed by TOPLESS (TPL)-like (SlTPL1) and HISTONE DEACETYLASE19 (HDA19)-like (SlHDA1) under the mediation of INHIBITOR OF MERISTEM ACTIVITY (SlIMA), and the complex can directly suppress *SlWUS* expression [24].

**Figure 1:**
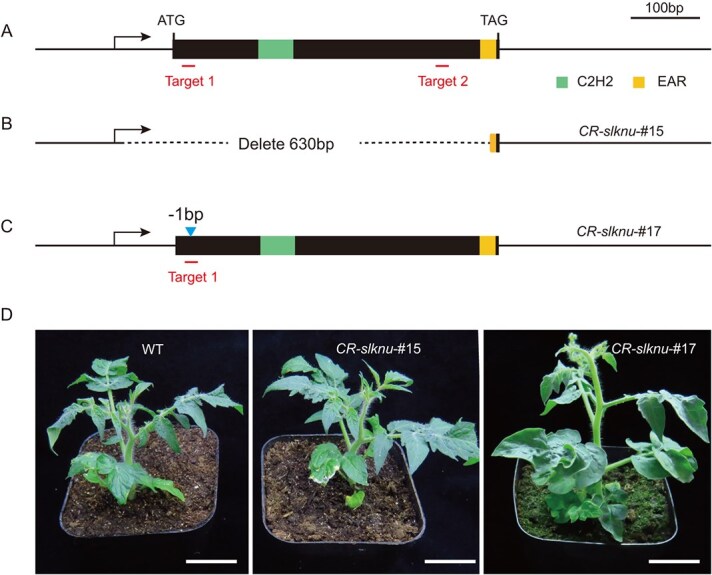
Construction of *SlKNU* gene knockout mutants. (**A**) Schematic representation of the *SlKNU* gene structure with exons indicated by black rectangles. (**B**) Schematic representation of CRISPR/Cas9-mediated *SlKNU* gene editing showing a deletion of 630 bp. (**C**) Schematic diagram of gene editing of CR-*slknu*-#17, with the editing sites marked by blue triangles. (**D**) Tomato seedlings edited by CRISPR/Cas9 exhibiting normal growth, scale bar = 3 cm.

In this study, we obtained knockout mutants of *SlKNU* by using CRISPR/Cas9. Mutants of CR-*slknu* showed enhanced FM activity, increased number of locules, and enlarged fruit size. Additionally, we found that SlKNU can directly bind to the promoters of both *SlCLV3* and *SlCLV1* and suppress their expression. Thus, SlKNU participates in FM determinacy control of tomato, thereby effectively regulating the size of tomato fruits. Our results also demonstrate that overexpression of *SlKNU* in *Arabidopsis* leads to dwarfism, slow growth, and loss of apical dominance, while the development of carpels is largely normal. Besides, in the background of *knu-2,* which is a null mutant of *Arabidopsis* [23], the floral defects are fully rescued by *SlKNU* driven by the endogenous *AtKNU* promoter. Hence *SlKNU* and *AtKNU* may have both functional conservation and divergence between the two species.

**Figure 2:**
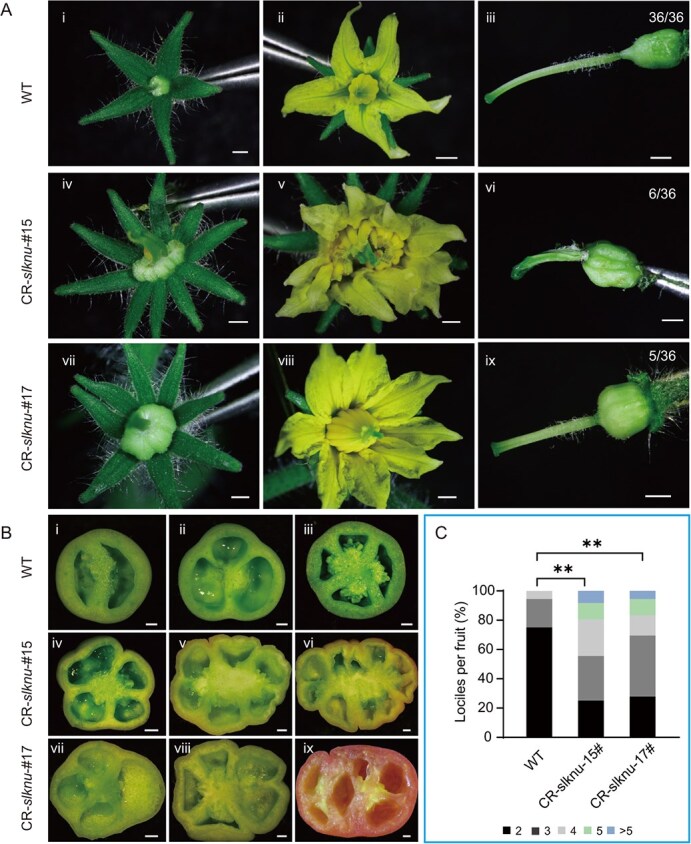
Phenotype of tomatoes of CR-*slknu* mutants. **(A)** Tomato floral organs. WT tomato floral organs include sepals (i), petals and stamens (ii), as well as pistils (iii). Flowers of CR-*slknu* mutants exhibit increased number of sepals (iv, vii), abnormal number of petals and stamens (v, viii), and complete pistil structures (vi, ix). Scale bar = 1 mm. **(B)** Cross-sections of tomato fruits. Cross-sections of WT tomato fruits at 34 days postanthesis (dpa) (i-iii). Cross-sections of CR-*slknu*-#15 tomato fruits at 40 dpa (iv–vi). Cross-sections of CR-*slknu*-#17 tomato fruits at 40 dpa (vii, viii) and 46 dpa (ix). Scale bar = 2 mm. **(C)** Statistics of locule numbers in CR-*slknu* mutants and WT tomatoes. (^**^P < 0.01, χ2 test, *n* = 36).

## Results

### Construction of *SlKNU* loss-of-function mutants via CRISPR/Cas9

SlKNU, a transcription factor with homology to *Arabidopsis* AtKNU, has been identified in tomato. SlKNU can directly suppress *SlWUS* in tomato, and SlKNU protein contains a conserved C2H2 zinc finger domain and a C-terminal EAR repressive domain [24]. To further investigate the function of SlKNU, we designed two single-guide RNAs (sgRNAs), and used CRISPR/Cas9 to gene-edit the specific regions of *SlKNU*, thereby aiming to generate *SlKNU* knockout mutants. The first target is situated 98 bp upstream of the conserved C2H2 domain, and the second target is 63 bp upstream of the EAR domain (Fig. 1a). Eventually we obtained 20 T1 transgenic tomato plants. Sequencing analysis revealed that five lines underwent editing at different sites, all presenting heterozygous mutations. We collected seeds from these plants and continued screening in the T2 generation. In the T2 generation, we successfully identified two homozygous mutant lines, namely CR-*slknu*-#15 and CR-*slknu*-#17. Notably, the CR-*slknu*-#15 line exhibited a large fragment deletion, resulting in the removal of a 630-bp DNA sequence (Fig. 1b). Compared to the WT, agarose gel electrophoresis of polymerase chain reaction (PCR) products revealed a significantly smaller band of *SlKNU* locus for CR-*slknu*-#15 (Supplementary Data Fig. S1a), which is confirmed by further sequencing analysis (Supplementary Data Fig. S1b). In the CR-*slknu*-#17 line, sequencing results revealed a single-base deletion that resulted in the precocious termination of protein translation with only six amino acids (Fig. 1c and Supplementary Data Fig. S1c). Despite this, the phenotype of the CR-*slknu*-#15 and CR-*slknu*-#17 did not show obvious vegetative growth defects compared to WT (Fig. 1d).

### SlKNU controls the size of the fruit

Both CR-*slknu*-#15 and CR-*slknu*-#17 exhibit various abnormal phenotypes in flower development. When comparing the number of floral organs between WT and CR-*slknu* mutants, we found a general increase in the latter. This increase was particularly noticeable in the number of sepals, petals, and stamens (Fig. 2a  **i–ii, iv–v, vii–viii)**. Compared to WT, where the average number of sepals is five, the mutants exhibit an average of approximately eight sepals. Similarly, while the average number of petals in the WT is five, in the CR-*slknu* mutants, it can reach up to 11, with an average of about seven. Additionally, the number of stamens in the WT usually ranges from five to six, whereas in the CR-*slknu* mutants, the average is approximately seven. (Supplementary Data Fig. S2 and Table S3). Besides, the ovary of CR-*slknu* mutants was significantly larger than that of the WT (Fig. 2a iii, vi, ix). Moreover, we also observed an increase in the number of locules in individual tomato fruits of CR-*slknu* mutants**,** consistent with the previously reported *35S:SlKNU-RNAi* fruits [24]. Cross-section of the fruits of WT tomato fruits (Fig. 2b  **i–iii)** and CR-*slknu* mutants (Fig. 2b  **iv–ix)** also showed that the number of locules in CR-*slknu* tomato fruits was obviously more than that in WT tomatoes. Statistical analysis revealed that, compared to the WT tomatoes, the proportion of fruits with more than three locules was significantly higher in the CR-*slknu*-#15 and CR-*slknu*-#17 mutants (Fig. 2c and Supplementary Data Table S3).

**Figure 3:**
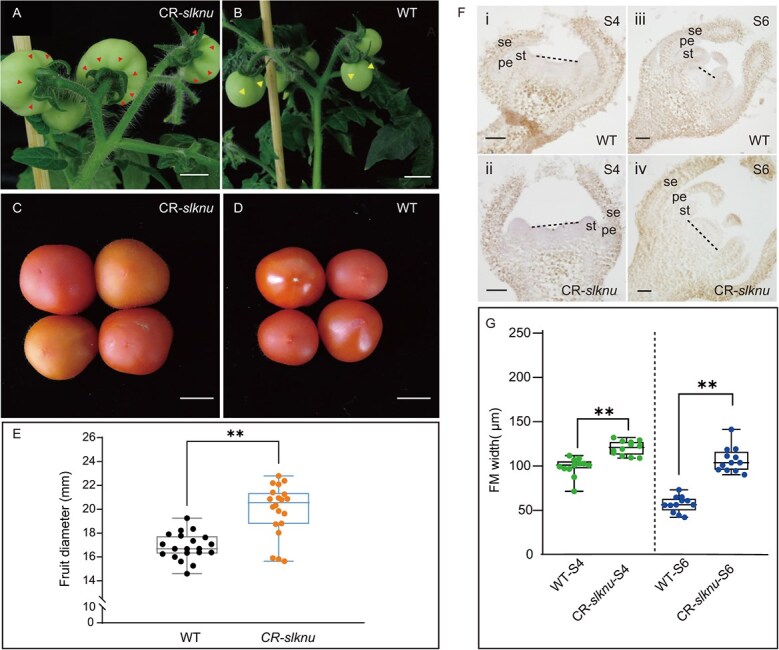
FM and fruit phenotype of CR-*slknu* plants. (**A**, **B**) Show the comparison of carpel numbers between CR-*slknu* (**A**) and WT (**B**). (**C**, **D**) Illustrate the size differences of fruits between CR-*slknu* (**C**) and WT (**D**). Scale bar = 1 cm. (**E**) Measurements of fruit diameters for both WT and CR-*slknu* mutant tomatoes at 46 dpa (*n* = 20 fruits). (**F**) FM width of WT tomato floral primordia at different developmental stages, with diameters marked by black dashed lines. WT at Stage S4 (**i**), CR-*slknu* at Stage S4 (**ii**), WT at Stage S6 (**iii**), and CR-*slknu* at Stage S6 (**iv**). Scale bars = 50 μm. ‘se’ denotes sepals, ‘pe’ denotes petals, and ‘st’ denotes stamens. **(H)** Quantitative analysis of FM width at Stages S4 and S6 for WT and CR-*slknu* (*n* = 12). Statistical significance between WT and CR-*slknu* mutants is indicated by asterisks. (^**^P < 0.01, ^***^P < 0.001, Student’s *t*-test).

In the CR-*slknu-#15* line, the large deletion of *SlKNU* CDS makes it a nearly null mutant. Therefore, CR-*slknu*-*#15* was chosen for further experiments and named as CR-*slknu*. We observed an increase in the number of carpels (Fig. 3a and b), resulting in a significantly larger size of mature CR-*slknu* fruit compared to WT (Fig. 3c and d), as indicated by the increased diameter of CR-*slknu* tomato fruits (Fig. 3e and Supplementary Data Table S3). The increase in the number of floral organs is primarily caused by the enlargement of the FM [25]. Thus, we measured the diameters of the FM of Stage 4 and Stage 6 flower buds in both WT and CR-*slknu* and found that the FM diameter in CR-*slknu* was significantly larger than that in the WT (Fig. 3f, g and Supplementary Data Table S3). Collectively, these results suggest that SlKNU regulates the activity of FM, thereby affecting the development of the carpel numbers and ultimately controlling fruit size.

**Figure 4:**
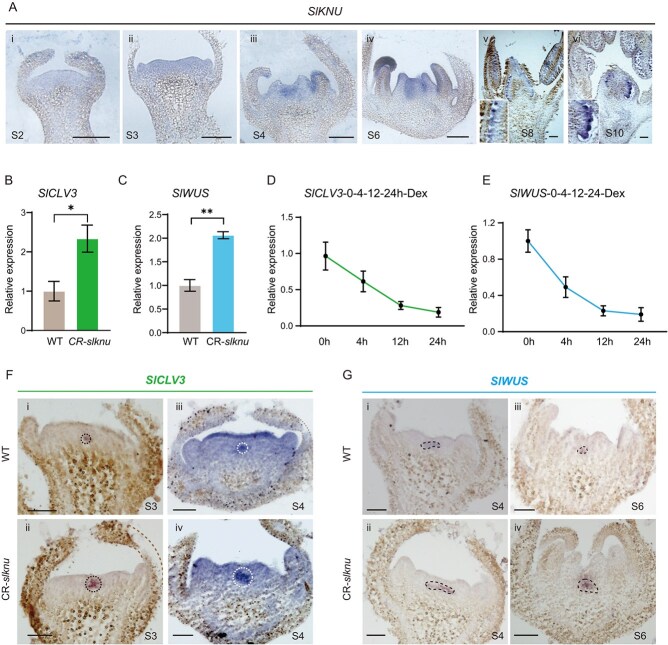
Repression of *SlCLV3* and *SlWUS* expression by SlKNU. **(A)**  *In situ* hybridization of *SlKNU* in WT flowers at stages 2 (i), 3 (ii), 4 (iii), 6 (iv), 8 (v), and 10 (vi). Scale bar = 100 μm. **(B, C)** Expression levels of *SlCLV3*  **(B)** and *SlWUS*  **(C)** in WT and CR-*slknu*, with error bars representing the standard error of three biological replicates. Asterisks indicate significant differences between WT and CR-*slknu*. **(D, E)** Dynamic expression changes of *SlCLV3*  **(D)** and *SlWUS*  **(E)** at various time points (0, 4, 12, 24 h) after DEX treatment,. Data are presented as mean ± standard error, with each time point representing three independent biological replicates. (^*^P < 0.05, ^**^P < 0.01, Student’s *t*-test). (**F, G**) In both WT and CR-*slknu*, *SlCLV3* expression is observed at Stage 3 **(F-i, ii)** and Stage 4 **(F-iii, iv)**; *SlWUS* expression is noted at Stage 4 **(G-i, ii)** and Stage 6 **(G-iii, iv)**. Dashed lines encircle or highlight the gene expression areas. Scale bar = 50 μm.

### SlKNU negatively regulates *SlCLV3* and *SlWUS*

The enlarged FM phenotype of CR-*slknu* resembles that of *fas* and *lc* mutants in tomato. [26]. By contrast, in *SlWUS-RNAi* tomato plants, the size of flowers was significantly reduced, and the number of locules in fruit also decreased [21]. These demonstrate that disruption of the *SlCLV3-SlWUS* negative feedback loop may lead to changes in tomato FM size.

*SlKNU* gene expression was first detected in the FM of Stage 3 flower buds and continued until Stage 20 in the carpels [24, 27]. However, *SlKNU* expression was not detected in the immature green fruit stage via RT-qPCR, nor it was observed in the subsequent mature fruit stages (Fig. 4a and Supplementary Data Fig. S3). Thus, we harvested inflorescences from WT and CR-*slknu* prior to Stage 8 and performed Real-time quantitative PCR (RT-qPCR) analysis for *SlCLV3* and *SlWUS* expression (Fig. 4b, c and Supplementary Data Table S2). The results demonstrated significant upregulation of both *SlWUS* and *SlCLV3* expression in CR-*slknu*. To investigate the regulatory role of SlKNU on *SlWUS* and *SlCLV3* expression, we generated *35S:SlKNU-GR-3xmyc* transgenic tomato lines, in which the SlKNU can be translocated from the cytoplasm to the nucleus upon dexamethasone (DEX) treatment. Twenty-four hours after initial DEX treatment, expression level of *SlKNU* in these lines was found to be >2000-fold higher than that of the WT (Supplementary Data Fig. S4 and Table S2). Four hours post-treatment, a sharp decline in both *SlCLV3* and *SlWUS* expression levels was observed. Suppression of *SlCLV3* and *SlWUS* by induced SlKNU persisted until 24 h (Fig. 4d, e and Supplementary Data Table S2), suggesting that SlKNU can strongly repress *SlWUS* and *SlCLV3* at transcription level. We also performed *in situ* hybridization assays to observe mRNA expression patterns of *SlWUS* and *SlCLV3* in CR-*slknu* mutant. In FMs of Stage 3–4 floral buds, the mRNA expression domains of *SlCLV3* (Fig. 4f) and *SlWUS* (Fig. 4g) both expanded in CR-*slknu* mutant compared to WT, but no evident alterations were observed during Stage 2 (Supplementary Data Fig. S5). Collectively, these results suggest that SlKNU suppresses *SlWUS* and *SlCLV3* expression, potentially mirroring the role of AtKNU in *Arabidopsis* by directly inhibiting *WUS* and *CLV3* to timely terminate FM activity and ensure proper carpel development [14, 23].

**Figure 5:**
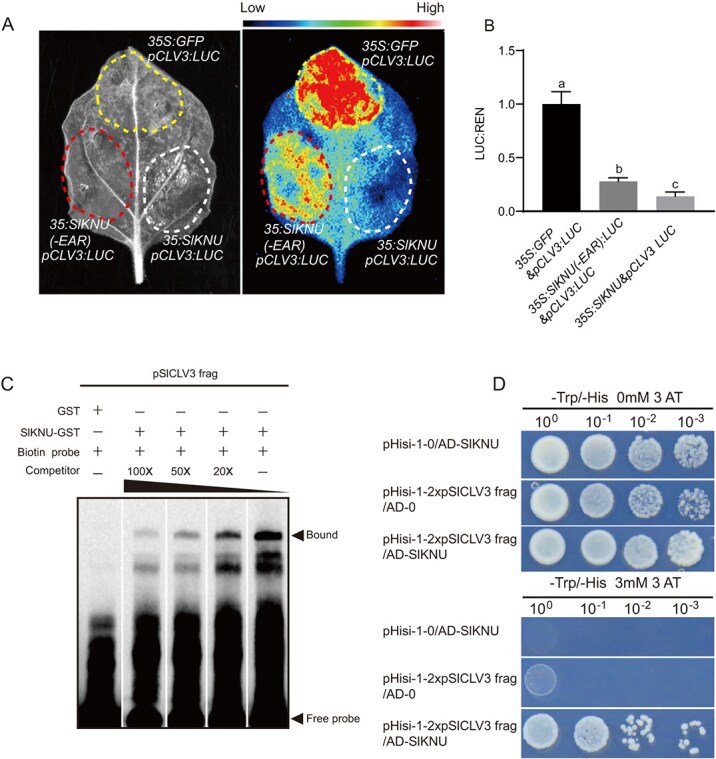
Direct repression of *SlCLV3* by SlKNU. (**A**) Dual-luciferase assays in *N. benthamiana* leaves of *pSlCLV3:LUC* coexpressed with *35S:eGFP* and *35S:SlKNU-eGFP*, and *35S:SlKNU-eGFP*. (**B**) Quantitative analysis of LUC expression by qRT-PCR. (**C**) EMSA confirmed that the GST-fused SlKNU protein binds to the sequence ‘TAATTTGACATAAAAAAATTAACTTTTAAATCTTATATCAAAAAA’, which is a fragment (#3) within the *SlCLV3* promoter. GST alone was used as the negative control. (**D**) Y1H analysis was conducted to detect the interaction between SlKNU and two tandem-repeated SlKNU-binding fragments of the *SlCLV3* promoter (named 2xpSlCLV3 frag). Transformed yeast cells were cultured on media lacking tryptophan and histidine (SD/−Trp/−His). pHisi-1–0 or AD–0 indicates the use of empty vectors. To suppress self-activation, 3 mM of 3AT (3-amino-1,2,4-triazole) was used. Statistical significance of the data in **(B)** was calculated using Student’s *t*-test, with bar graphs representing mean ± standard error from three biological replicates. Different lowercase letters (‘a’, ‘b’, ‘c’) indicate statistically significant differences between groups.

### SlKNU directly represses *SlCLV3* expression

To test if SlKNU can directly repress *SlCLV3*, we performed dual-luciferase assays in tobacco. A luciferase reporter gene vector driven by the *SlCLV3* promoter, *pCLV3:LUC*, and a 35S promoter-driven *SlKNU-eGFP* fusion protein, *35S:SlKNU-eGFP*, were constructed. Given that the SlKNU contains two functional domains, an N-terminal C2H2 domain and a C-terminal EAR domain, which plays a major role in transcriptional repression [28], we also generated a line for the overexpression of SlKNU lacking the EAR domain, named *35S:SlKNU-eGFP*, to assess the requirement of the EAR domain for SlKNU function. In tobacco leaves, the luciferase (LUC) signal was obvious in the control group of *35S:eGFP* + *pCLV3:LUC*. Compared to the *35S:eGFP* + *pCLV3:LUC* group, the LUC signal in the *35S:SlKNU-eGFP* + *pCLV3:LUC* group was significantly weakened and almost undetectable, while the LUC signal in the *35S:SlKNU-eGFP* + *pCLV3:LUC* group was weaker but still observable (Fig. 5a). These results suggest that SlKNU directly repress *SlCLV3* promoter activity partially through its EAR domain. In addition, the quantitative assays by qRT-PCR were consistent with the LUC signals (Fig. 5b and Supplementary Data Table S2). To verify whether SlKNU could directly bind to the *SlCLV3* promoter and repress its expression, we performed an electrophoretic mobility shift assay (EMSA) *in vitro*. Based on the binding preference of the AtKNU to the reported ‘AACTNT’ sequence [14], we analyzed the *SlCLV3* promoter region and identified three putative SlKNU binding sites located at −260 to −305 bp (#1), −1082 to −1127 bp (#2), and − 1988 to −2033 bp (#3). The EMSA results showed no specific binding between SlKNU and Fragments #1 and #2 (Supplementary Data Fig. S6a). However, a clear band was observed between SlKNU and Fragment #3. Competitive binding assays with increasing concentrations of unlabeled cold probes showed gradually weakened signals, confirming the specific interaction between SlKNU and Fragment #3 of *SlCLV3* promoter (Fig. 5c). Additionally, the binding of SlKNU to the *SlCLV3* promoter was also verified by the yeast one-hybrid (Y1H) assay (Fig. 5d). Given that in *Arabidopsis*, AtKNU not only inhibits the expression of *CLV3* but also directly inhibits *CLV1* [14], we further investigated whether SlKNU in tomato could also directly represses the expression of *SlCLV1*. In tobacco leaves, the downregulation of *SlCLV1* by SlKNU is similar to that of *SlCLV3* (Fig. 5 and Supplementary Data Fig. S7a–b). Additionally, we identified sequences containing the ‘AACTNT’ motif within the promoter region of *SCLV1* and selected two potential SlKNU binding sites located at −1452 bp to −1407 bp (#1) and −924 bp to −879 bp (#2). EMSA revealed that SlKNU could only bind to Site #2，but not Site #1 (Supplementary Data Fig. S6b and S7c). Therefore, SlKNU can directly bind to the promoters of both *SlCLV3* and *SlCLV1*, participating in the repression of both *SlCLV3* and *SlCLV1*. This further illustrates the functional conservation between SlKNU and AtKNU.

We also examined whether SlKNU could also directly repress the expression of the closest paralog of *SlCLV3*, *SlCLE9*, which may act as an active compensatory factor in tomato when *SlCLV3* expression is compromised [16]. For this purpose, we coinjected *35S:SlKNU-eGFP* with *pCLE9-LUC* into tobacco leaves using the dual-luciferase reporter system. There is no significant difference between the *35S:SlKNU-eGFP* + *pCLE9-LUC* group and the *35S:eGFP* + *pCLE9-LUC* control group (Supplementary Data Fig. S6c–d and Table S2). These suggest that SlKNU specifically inhibits *SlCLV1* and *SlCLV3* but SlKNU does not directly inhibit *SlCLE9*.

### SlKNU and AtKNU exhibit conserved functions

**Figure 6:**
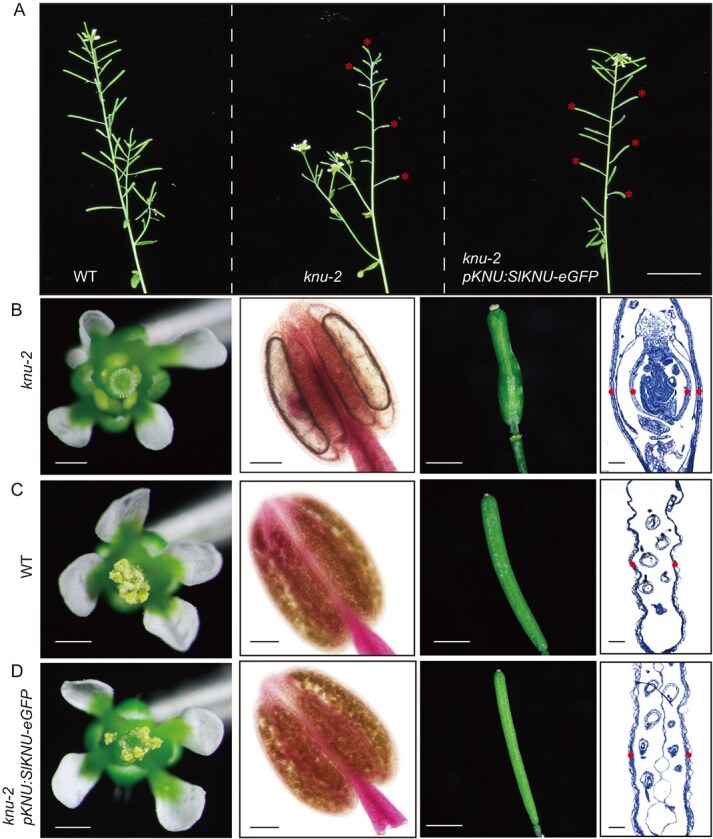
*pKNU:SlKNU-eGFP* fully rescues the floral phenotype of *knu-2*. (**A**) Overall morphology of WT, *knu-2*, and *knu-2 pKNU-SlKNU-eGFP* plants. Red asterisks indicate siliques. Scale bar = 2 cm. (**B–D**) Flowers, anthers, siliques, and longitudinal sections of siliques from *knu-2* (**B**), WT (**C**) and *knu-2 pKNU: SlKNU-eGFP* (**D**). Silique sections were stained with 0.1% toluidine blue in 0.02% sodium carbonate solution. Red dots indicate the position of carpels. Scale bars are 500 μm for flowers and siliques and 100 μm for anthers and longitudinal sections of siliques.

SlKNU and AtKNU both retained the C2H2-type zinc finger and EAR repressor domains, which suggests their functional similarities [24]. To test this, we generated *35S:SlKNU-eGFP* in *Arabidopsis* and obtained 30 T1 transgenic plants, whose phenotypes can be categorized into three groups as strong (33%), moderate (53%), and weak (14%) (Supplementary Data Fig. S8a–c and Table S3). This variation may be attributed to different expression levels of the SlKNU protein caused by positional effects of the transgenes. Compared to WT (Supplementary Data Fig. S8d), for the strong group, plants exhibited loss of apical dominance, slow growth, inability to bolt, narrow leaves, and partial waxiness (Supplementary Data Fig. S8e). For the moderate group, plants showed weakened apical meristem activity, stunted growth, and late flowering (Supplementary Data Fig. S8f and g). In the flowers of the moderate group of *35S:SlKNU-eGFP*, the stamen number decreased compared to the WT, whereas there were no significant changes in the number of other floral organs (Supplementary Data Fig. S9a–l and Table S3). Unlike dihydrotestosterone (DHT)-treated *35S:AtKNU-AR Arabidopsis* plants, which showed carpel-less flowers [29], *35S:SlKNU-eGFP* flowers still produce carpels (Supplementary Data Fig. S9k), while the seed-setting rate of *35S:SlKNU-eGFP* was significantly reduced. This may be partially attributed to the significant reduction of pollen grains in the flowers of *35S:SlKNU-eGFP* (Supplementary Data Fig. S9m and n). To further assess the functional conservation between SlKNU and AtKNU, we expressed *SlKNU* driven by the *AtKNU* promoter in *knu-2* background. Given the specific expression of *AtKNU* in megaspore mother cells, we detected GFP fluorescence in the megaspore mother cells of *knu-2 pKNU:SlKNU-eGFP* (Supplementary Data Fig. S10). Then we observed the phenotype of *knu-2 pKNU:SlKNU-eGFP* plants (Fig. 6a) and their flowers, and performed Alexander staining of the anthers as well as longitudinal sectioning of the pistils (Fig. 6b–d). We found that unlike the null mutant *knu-2* anthers lacking pollen and *knu-2* pistils with ectopic internal carpels [23] (Fig. 6b), *knu-2 pKNU:SlKNU-eGFP* flowers resemble WT flowers and show fully rescued floral phenotypes (Fig. 6c, d). These results indicate that specific expression of *SlKNU* driven by *AtKNU* promoter in the *knu-2* background can fully rescue the *knu-2* floral defects.

## Discussion

The conserved *CLV3-WUS* feedback loop functions to maintain SAM and FM activity in *Arabidopsis*, tomato, and some other plant species [30]. During early stages of tomato floral development, FM activity is crucial for generating sufficient number of cells to differentiate into various floral organs. Alterations in *CLV3-WUS* pathway can lead to changes in meristem size [17]. The size of tomato fruit is primarily determined by the number of locules in mature fruit [31], which is closely regulated by the *CLV3-WUS* pathway [32]. Besides, a member of the AP2/ERF superfamily, EXCESSIVE NUMBER OF FLORAL ORGANS (ENO), represses the expression of *SlWUS* to limit fruit size of tomato [26].

In our study, we found that SlKNU can directly suppress *SlCLV3* and *SlCLV1*, thereby controlling FM activity to limit the number of carpels and fruit size (Fig. 2). YABBY family proteins CRABS CLAW a (SlCRCa) and SlCRCb also function to regulate tomato FM activity, and *slcrca* and *slcrcb* mutants both display delayed FM termination which leads to fruit-within-fruit phenotype [27]. SlCRCb can directly bind to the second intron region of *SlWUS*, thereby directly suppressing *SlWUS* [33], Epigenetic regulatory factors also play a role in the regulation of tomato meristematic tissue activity. Recent studies have found that reduced activity of the histone acetyltransferase SlGCN5 leads to dwarfism in tomato plants with decreased *SlWUS* expression, and obviously reduced SAM and FM size [34].

Here we observed that the FM size of CR-*slknu* is significantly enlarged compared to WT, showing the enhanced FM activity in CR-*slknu* (Fig. 3). It has been reported that SlKNU can recruit the histone deacetylase SlHDA1 to the chromatin of *SlWUS*, thereby participating in the epigenetic repression of *SlWUS* [24]. In *Arabidopsis*, AtKNU can directly suppress the expression of *CLV1*, *CLV3*, and *WUS* [14, 23]. Therefore, we also verified through EMSA that SlKNU can specifically bind to the promoter regions of *SlCLV3* and *SlCLV1* (Fig. 5 and Supplementary Data Fig. S7). This suggests that SlKNU and AtKNU play conserved and important roles in the development of flowers in different plant species.

Despite research in tobacco leaves showing that SlKNU-EAR still exhibits some repressive activity on the promoters *pSlCLV1* and *pSlCLV3*，the intensity of suppression is reduced compared to the full-length SlKNU. This result indicates that the EAR domain plays a crucial role in the suppressive function of SlKNU. In *Arabidopsis*, transcription factors containing the EAR domain are known to recruit transcriptional repression complexes, such as histone deacetylase complexes and Polycomb group (PcG) proteins, mediating H3 deacetylation and H3K27me3 formation, thereby suppressing downstream genes expression [28]. Additionally, in *Arabidopsis*, AtKNU can interact with WUS protein, disrupting the formation of WUS-WUS dimers, an interaction that does not depend on the EAR domain. This KNU-WUS interaction can also inhibit the binding of WUS to the *CLV3* promoter, thereby preventing the activation of *CLV3* expression [14]. Thus, in tomato, SlKNU might similarly interact with SlWUS protein, hindering the binding of SlWUS to the *SlCLV3* locus and indirectly repressing the expression of *SlCLV3*. In eukaryotic cells, RNA polymerase II is responsible for the transcription of DNA into mRNA [35]. SlKNU might directly bind to the proximal promoters of the downstream target genes, thereby preventing the binding of RNA polymerase II. The absence of the EAR domain might weaken this capability, leading to diminished gene suppression. Future research should further explore how SlKNU precisely regulates gene expression through these mechanisms for understanding the SlKNU-mediated regulatory networks in plant development.

In tomato, high-temperature treatment inhibits the synthesis of brassinosteroids (BR), which may deactivate *CRABS CLAW a* (*SlCRCa*), thus leading to upregulation of *SlWUS* [33]. This results in a phenotype of fruit-within-fruit, resembling *slcrca* mutants [27]. In *Arabidopsis*, CRC and KNU are both directly activated by AGAMOUS(AG) [29, 36, 37]. Whereas in tomato, activation of *SlKNU* requires the combined action of both tomato AGAMOUS1 (TAG1) and SlCRCa [33], suggesting that the activation of SlKNU in tomato is also indirectly influenced by BR signaling.

In *Arabidopsis*, *knu* mutant shows delayed termination of *WUS*, leading to indeterminate FM resulting in ectopic carpels within the primary carpel [23], resembling the tomato *slcrc* mutant fruit phenotype [27, 33]. In tomato, CR-*slknu* mutants don’t show obvious vegetative growth defects and have fertile stamens. However, they produce enlarged fruits due to increased locule numbers, with no ectopic fruits appearing inside the original fruit, resembling the fruit phenotype of *slclv3* [38] (Fig. 3). In addition, AtKNU expression in *Arabidopsis* begins from Stages 5 to 6 of flower development [23, 39], when carpel primordia arises.

In our study, we found that *SlKNU* expression can be detected from Stage 3 onward [20, 27] of tomato floral development (Fig. 4), while *SlWUS* expression persists until Stage 7 [20]. Therefore, in *Arabidopsis*, AtKNU functions to terminate *WUS* activity to ensure proper carpel development. In tomato, SlKNU may begin to suppress *SlWUS* from floral Stage 3 onwards, and the termination of meristem activity might require the synergistic effects of SlKNU and SlCRCs [27, 33]. In DHT-treated *35S:KNU-AR* plants with induced KNU overexpression, carpel-less flowers and reduced stamen numbers were observed [29]. Thus, overexpression of AtKNU in *Arabidopsis* prematurely terminates the activity of the FM. However, overexpression of *SlKNU* in tomato does not completely terminate the activity of the FM. Studies have shown that tomatoes overexpressing *SlKNU* primarily exhibit a loss of apical dominance leading to a bushy growth during the vegetative growth phase. Although the floral organs are reduced in size, carpels are still produced, and the fruits developed later are noticeably smaller, indicating significant suppression of the FM activity, leading to changes in the size of floral organs and fruits [24]. Therefore, we overexpressed *SlKNU* from tomato in *Arabidopsis* and observed phenotypes showing loss of apical dominance and bushy growth (Supplementary Data Fig. S8d–f), similar to those seen in tomato overexpressing *SlKNU*, as well as noticeably weakened shoot apices, indicating that SlKNU can also suppress meristematic activity in *Arabidopsis*. Additionally, we observed a slight reduction in the number of stamens in the floral organs, but carpels are largely normal (Supplementary Data Fig. S9), unlike that *AtKNU* overexpression in *Arabidopsis* leads to carpel-less flowers [29]. Although SlKNU can substitute AtKNU function in *Arabidopsis*, thereby rescuing the *knu-2* (Fig. 6d), the activity of SlKNU in *Arabidopsis* may be slightly different from the native AtKNU. This suggests that although the functions of the two KNU genes are similar across these two species, they are not completely identical in terms of expression levels, roles in their respective regulatory networks, or specific interactions with other proteins.

## Conclusion

In summary, we explored the function of SlKNU in FM regulation in tomatoes. Knockout mutant of *SlKNU* generated by CRISPR/Cas9 exhibited enhanced FM activity, increased locule numbers, and enlarged fruit size (Fig. 2). Additionally, we discovered that SlKNU can repress both *SlCLV1, SlCLV3*, and *SlWUS* similarly as the role of AtKNU in *Arabidopsis*. In *Arabidopsis knu-2* mutant, expression of SlKNU driven by the *AtKNU* promoter can fully rescue the mutant phenotype (Fig. 6), further confirming the functional conservation between AtKNU and SlKNU. These findings not only contribute to the understanding of the molecular mechanisms of FM regulation in tomato but also provide a theoretical basis for potential yield enhancement of tomato.

## Materials and methods

### Generation of transgenic plants and chemical treatments

*Arabidopsis* transformation was carried out using the *Agrobacterium tumefaciens* (GV3101)-mediated floral dip method, as described previously [40]. Tomato transformation was conducted using the method reported in previous studies [41]. During the transformation process, tomato plants germinated for 10–12 days were selected, the tips of the cotyledons were removed, and the remaining parts were bisected and placed on MS medium plates for preculture. After 2–3 days of preculture, the cotyledons were soaked in the dark for 10 min in a suspension of GV3101, followed by a 48-h culture on MS medium in the dark. The cotyledons were then transferred to callus-inducing medium until callus formation occurred, and subsequently to shoot-inducing medium until new shoots developed. Ultimately, the plants were transferred to rooting medium to promote root development and then transplanted into soil to continue growth at room temperature. Transgenic plants containing *35S:SlKNU-eGFP* and *35S:SlKNU-GR-3xmyc* constructs were developed using the pGreen II vector. Mutants of the CR-*slknu* were generated using CRISPR/Cas9, following the related report [42]. Two 20-bp-specific sgRNAs targeting the exon region of the *SlKNU* gene were designed, driven by the U6 promoter, and cloned into the pHEE401 vector. Target sites in the T0 generation plants were amplified by PCR, and mutations at these sites were screened through PCR amplification and sequencing analysis. In the T1 generation, homozygous mutants were identified through further analysis of the target sites. The sequences of the specific primers used are available in the Supplementary Data Table S1.

DEX (BBI, A601187) treatment was carried out by inverting the plants and submerging the inflorescences in a 10-mM DEX solution, as previously described [23]. The commencement of the DEX treatment was designated as 0 h.

### RNA extraction and qRT-PCR analysis

Total RNA was extracted from tomato inflorescence samples using the RNA isolator Total RNA Extraction Reagent Kit (Vazyme, R401–01), according to the manufacturer’s protocol. Following extraction, 1 μg of total RNA was reverse-transcribed using HiScript II Q RT SuperMix (Vazyme, R323–01). To quantify the expression levels of target genes, we used ChamQ Universal SYBR qPCR Master Mix (Vazyme, Q711–03). The qPCR reactions were performed on the Step One Plus Real-Time PCR System from Applied Biosystems, with *SlACTIN2* serving as the internal control [34]. Each sample was subjected to three replicates to ensure the accuracy and reproducibility of the data. The sequences of the specific primers used are detailed in Supplementary Data Table S1.

### Electrophoretic mobility shift assay

EMSAs were performed in accordance with the methods previously reported [23]. The coding sequence (CDS) of the *SlKNU* was cloned into the EcoRI-digested pGEX-4 T-1 expression vector, enabling the fusion of the SlKNU protein with the glutathione S-transferase (GST) tag to produce a recombinant fusion protein. This construct was then transformed into the *Escherichia coli* Rosetta strain to express the SlKNU-GST fusion protein. The Rosetta strain was chosen for its enhanced expression and solubility of heterologous proteins. The fusion protein was purified using Glutathione resin, following the manufacturer’s guidelines, which allowed for the high-purity isolation of the target protein via affinity chromatography. Prior to EMSA, DNA probes were biotin-labeled and annealed by heating and slowly cooling double-stranded DNA to facilitate correct base pairing of complementary strands. The binding reactions were conducted using an EMSA kit (Thermo Scientific, 20 148) to detect the interactions between the protein and the biotin-labeled probes. Detailed information regarding the individual probes used is provided in Supplementary Data Table S1**.**

### *In situ* hybridization

The *in situ* hybridization assay was conducted according to the methods previously described [23]. Initially, DNA fragments corresponding to the target genes *SlCLV3*, *SlWUS*, and *SlKNU* were amplified from plant cDNA via reverse transcription PCR (RT-PCR), utilizing primer sequences from Supplementary Data Table S1. These PCR products were subsequently cloned into the pGEM-T Easy vector (TIANGEN, VT307) and linearized for *in vitro* transcription. Utilized for the *in vitro* transcription was the DIG RNA Labeling Kit (Roche, 11 175 025 910). The results of the hybridization were examined and photographed using an Olympus BX53 microscope.

### Floral meristem size measurement

The size of the FMs was measured following the previously described methods [20, 34]. Floral buds of WT and CR-*slknu* mutants at Stages 4 and 6 were fixed in paraffin and prepared into 7- to 10-μm-thick sections according to methods previously described [23]. Subsequently, images were captured using a microscope, and data processing was conducted using ImageJ software. The width of the FMs was measured along the line between two stamen primordia within the bud. For each genotype and time point, at least 12 meristems were measured. Finally, statistical analysis was performed using a *t*-test to assess the significance of the data differences.

### Dual-luciferase assays

In *Nicotiana benthamiana*, a transient expression assay was conducted, as previously described [43]. To explore the potential inhibitory effects of SlKNU on *SlCLV3* and *SlCLE9*, the 2500 bp sequences upstream of the translation start sites of these two genes were cloned as promoters to drive the LUC reporter, resulting in the construction of *pSlCLV3:LUC* and *pSlCLE9:LUC* reporter gene vectors. *pSlCLE9:LUC* with *35S:eGFP* and *pSlCLV3:LUC* with *35S:eGFP* served as negative controls. The activities of Firefly and Renilla luciferases were quantitatively analyzed using the Dual-Luciferase Reporter Assay System [44]. The fluorescent regions in *N. benthamiana* leaves were imaged using the Bio-Rad ChemiDoc™XRS+ imaging system, with a color gradient visually indicating the variance in LUC luminescence intensity.

### Y1H assays

The experimental procedures were conducted according to the manufacturer’s instructions (Clontech). Initially, two tandem-repeated SlKNU-binding fragments were cloned into the pHisi-1 vector. Subsequently, the resulting constructs were linearized using Xho I enzyme (NEB, R0146V) and transformed into the Y1H Gold yeast strain provided by Weidi Biotech. The full coding sequence (CDS) of SlKNU was fused with the GAL4 activation domain in the pDEST22 vector, generating AD-SlKNU. Finally, the plasmid containing AD-SlKNU was transformed into the Y1H Gold strain harboring the corresponding pHisi-1 constructs.

### Alexander staining assay

To assess pollen viability, anthers from Stage 12 *Arabidopsis* flowers were collected and stained using Alexander’s solution as previously described [45]. Subsequently, images were captured using an OLYMPUS microscope (BX53F).

## Supplementary Material

Web_Material_uhae331

## Data Availability

Data supporting the findings of this work are available within the article/supplementary files. The plant materials and datasets generated and analyzed during the study are available from the corresponding authors upon reasonable request.
