# Supplementary material for: SlKNUCKLES regulates floral meristem activity and controls fruit size in Solanum lycopersicum
Source: Hortic Res. 2024 Nov 21;12(3):uhae331. doi: 10.1093/hr/uhae331 (PMC11879652; doi:10.1093/hr/uhae331)
Supplement: Web_Material_uhae331 [file web_material_uhae331.zip › HR-Supplemental Figures-2024-11-4.docx]

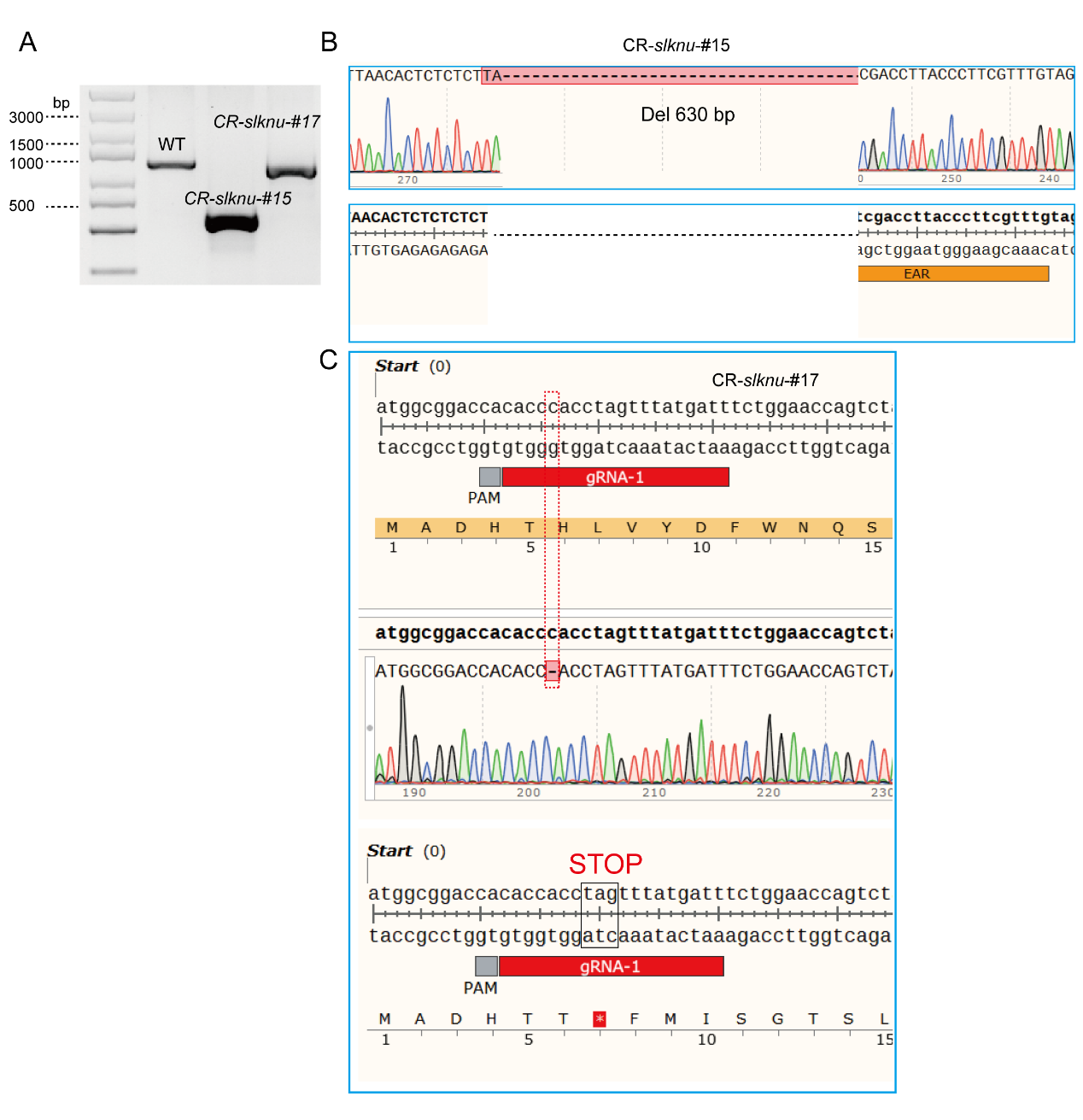


**Fig S1.** Analysis of sequencing results for *SlKNU* edited by CRISPR/Cas9. **(A)** Detection of CRISPR/Cas9-mediated gene editing in transgenic tomatoes using PCR. **(B-C)** Sequencing chromatograms of CR-*slknu*-#15 **(B)** and CR-*slknu*-#17 **(C)**, with the red regions indicating the deleted fragments.


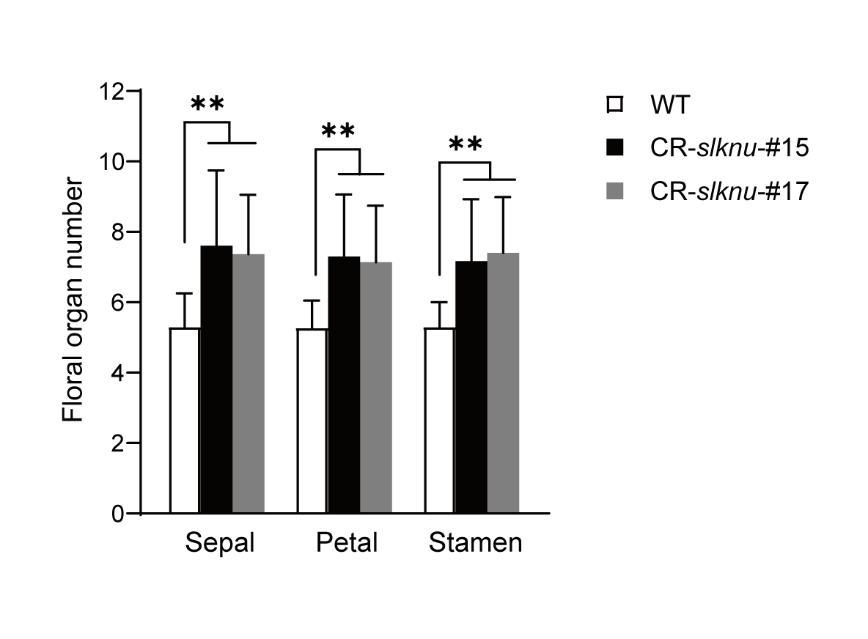


**Fig S2**. Quantitative analysis of floral organ numbers was conducted for both WT and CR-*slknu* mutants (n=30). (***p* < 0.01, Student’s t-test).


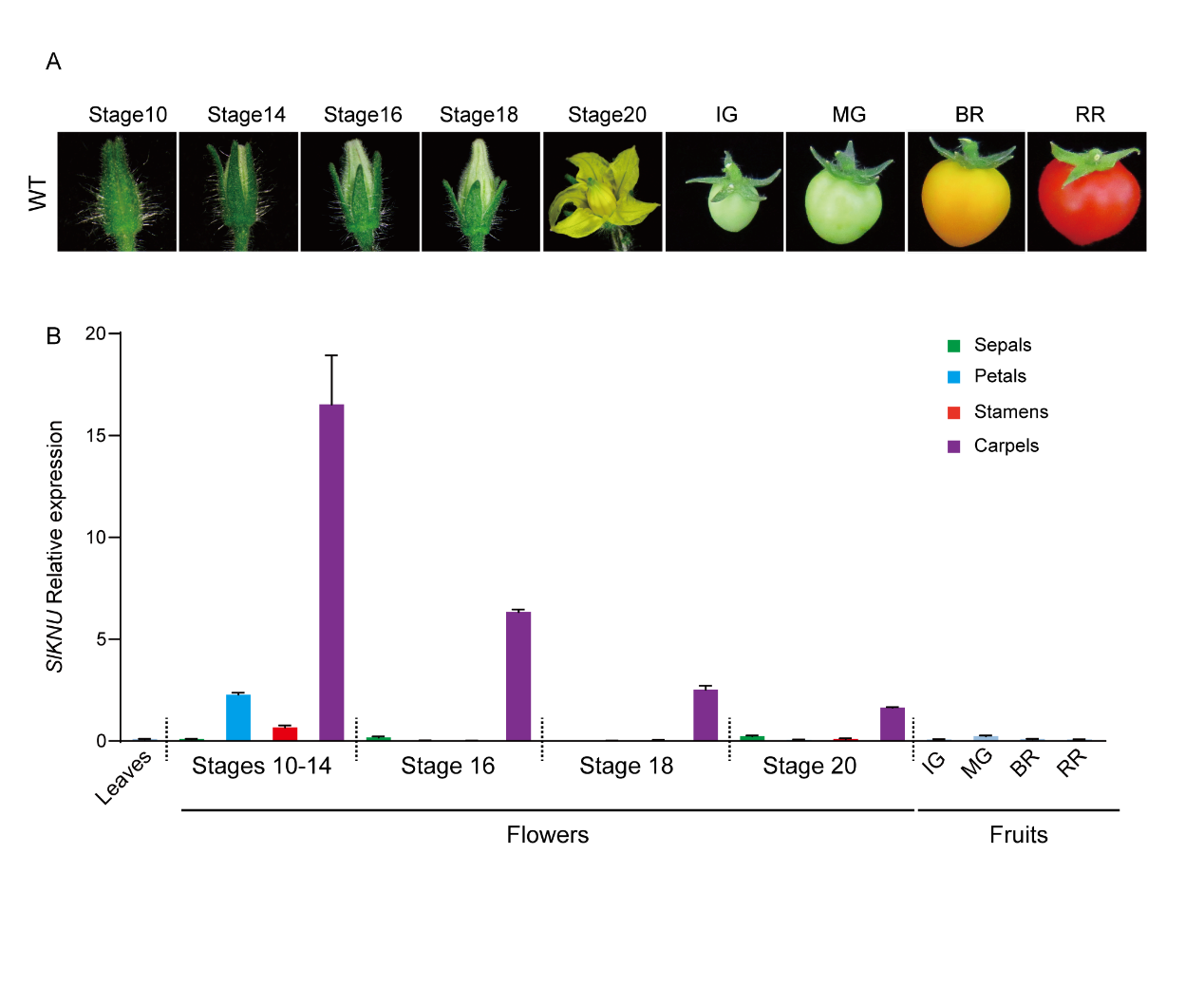


**Fig S3**. Dynamic expression levels of the *SlKNU* in tomato. **(A)** Different developmental stages of WT flowers and fruits. **(B)** Relative expression of *SlKNU* in various developmental tissues at different stages determined by qRT-PCR. IG represents immature green fruit; MG represents mature green fruit; BR represents breaker stage fruit; RR represents ripe red fruit. Data are mean ± standard error of three biological replicates.


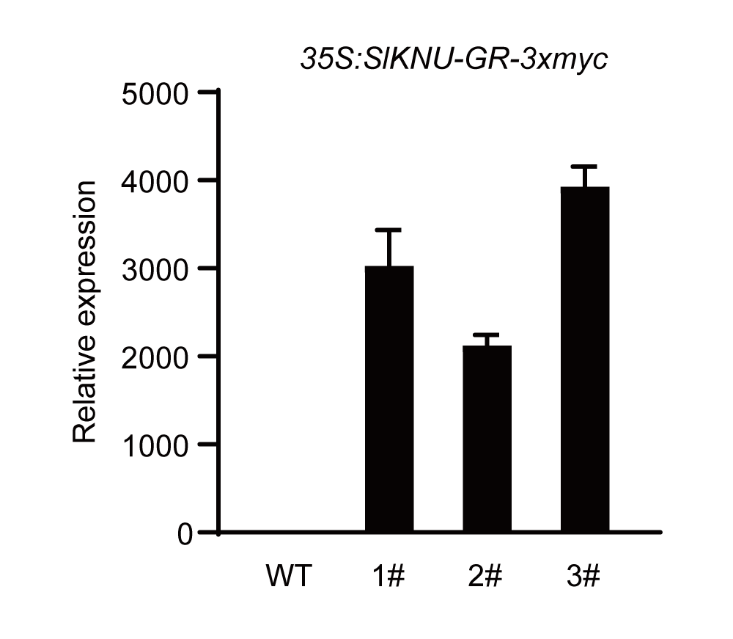


**Fig S4.** Identification of *35S:SlKNU-GR-3xmyc* Transgenic Tomatoes. Expression levels of *SlKNU* in transgenic tomato plants measured by qRT-PCR, #1, #2, and #3 indicated three different transgenic lines. Bars represent mean ± SE (n = 3, biological replicates).


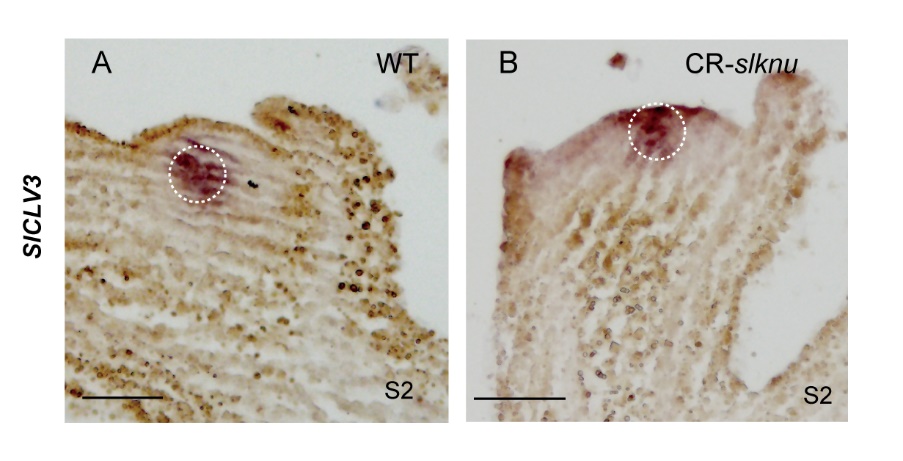


**Fig S5**. In situ hybridization demonstrated the expression of *SlCLV3* in stage 2 flowers of both the WT（A）and the CR-*slknu* (B). Scale bar=50 µm.


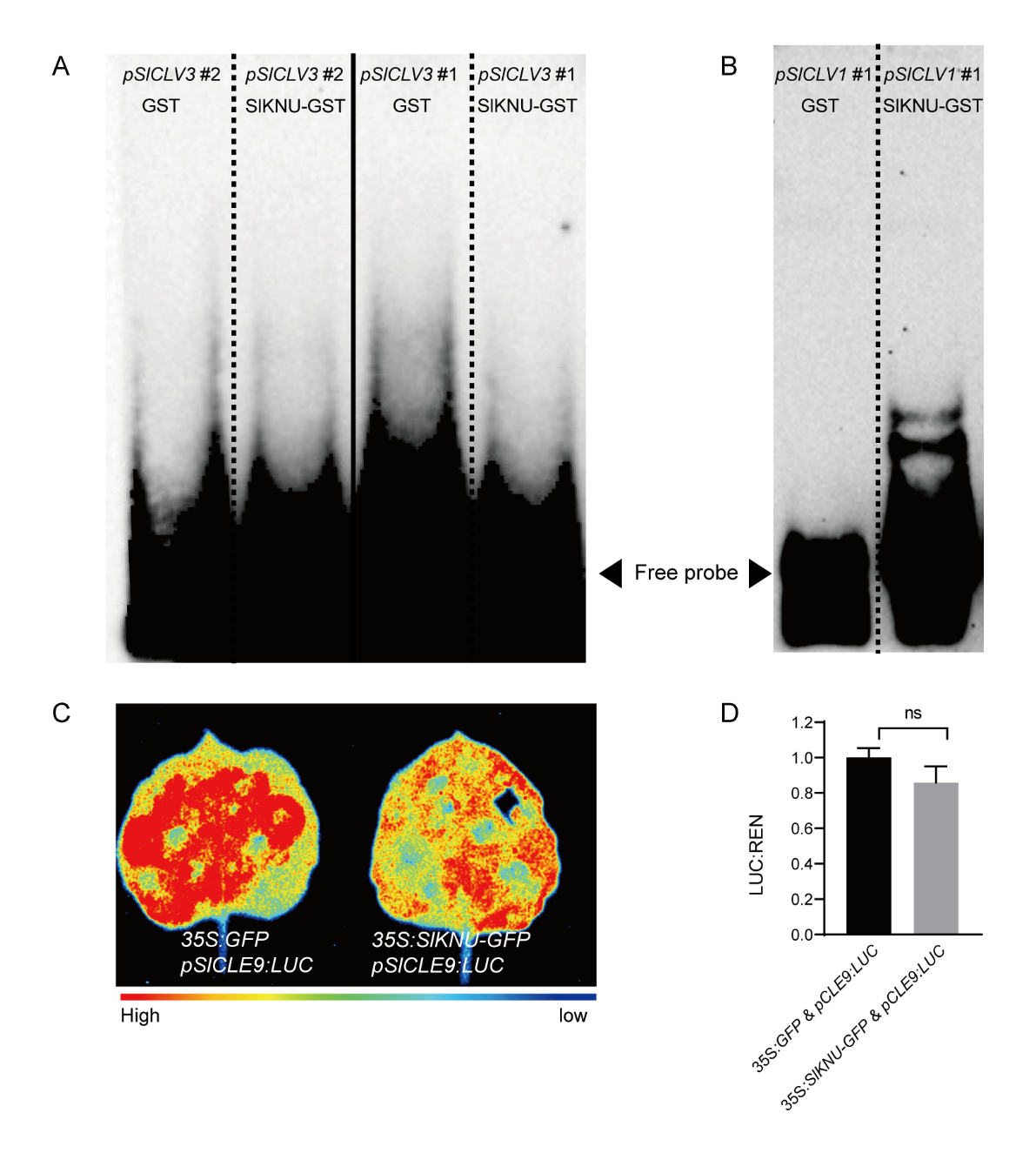


**Fig S6.** Test of protein binding to the promoters of *SlCLV1、SlCLV3* and *SlCLE9*. (**A,B)** The results of EMSA conducted to analyze the interactions between SlKNU protein and two potential fragments within the *SlCLV3* promoter, designated as #1 and #2**(A)**, as well as with the *SlCLV1* promoter, designated as *pCLV1*#1**(B)**. GST protein served as a negative control. (**C**) Dual-luciferase assay comparing the luciferase activity in leaves harboring the *pSlCLE9:LUC* co-expressed with *35S::GFP* (control) and *35S:SlKNU-eGFP* (test).(**D**) Quantitative analysis of luciferase expression by qRT-PCR. The P-value was generated by Student’s t-test. Bars represent mean ± SE (n = 3, biological replicates).


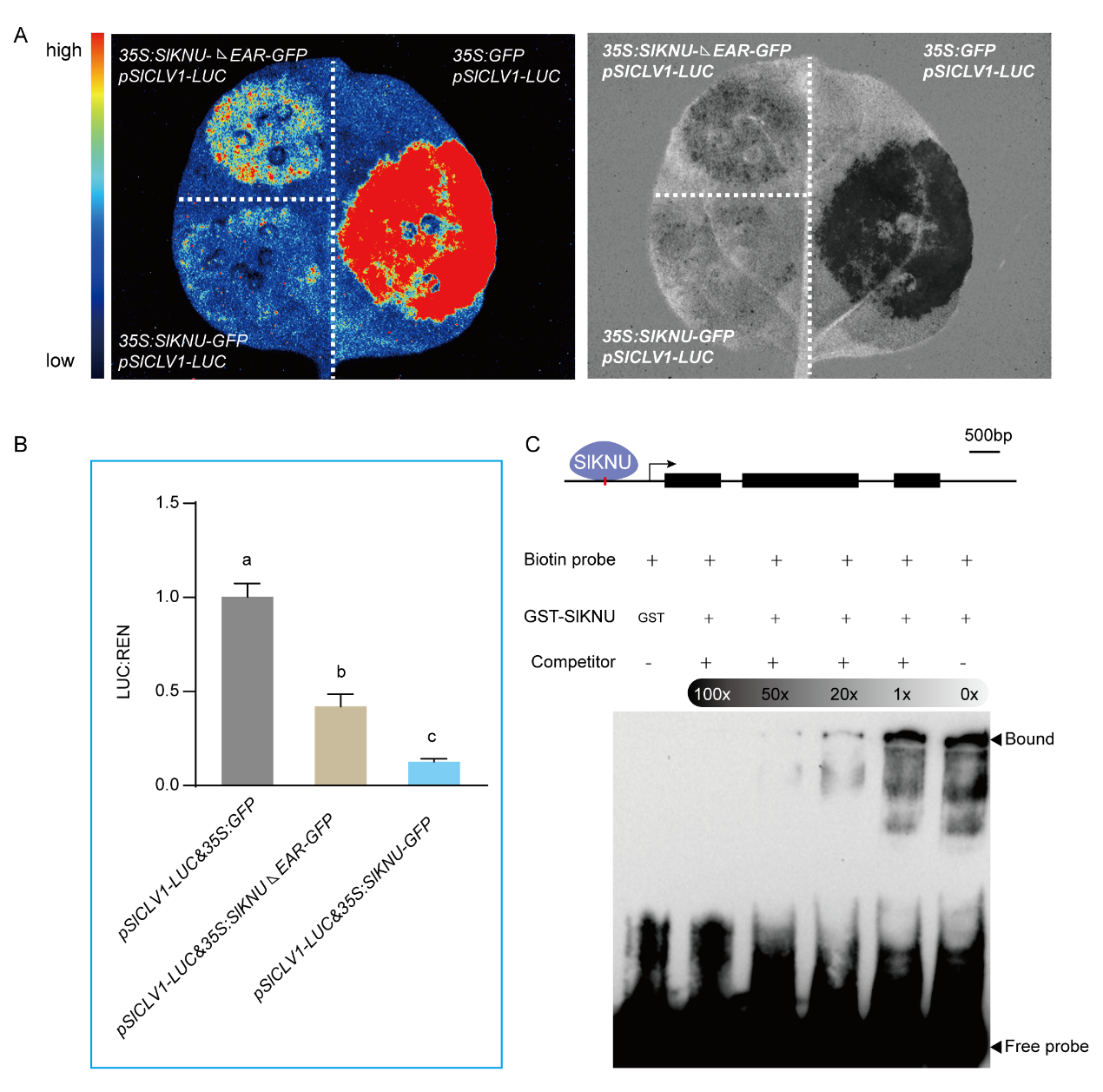


**Fig S7.** SlKNU inhibits the expression of *SlCLV1*. **(A)** In tabaco leaves, the effects of *pSlCLV1:LUC* with *35S:eGFP*, *35S:SlKNU-eGFP* and 35S:SlKNU◺-eGFP were compared using a dual-luciferase reporter assay system. **(B)** The expression of luciferase was quantitatively analyzed by real-time qRT-PCR. Statistical significance was calculated using Student's t-test, and the data are presented as the mean ± standard error of three biological replicates. Different lowercase letters ('a', 'b', 'c') indicate statistically significant differences between groups. **(C)** EMSA confirmed that the GST-fused SlKNU protein can bind to the sequence "ACAAAGAGATATACATAATAAACTATTTTGAAATAATCCAAAAATA" within the *SlCLV1* promoter (labeled as fragment #2).


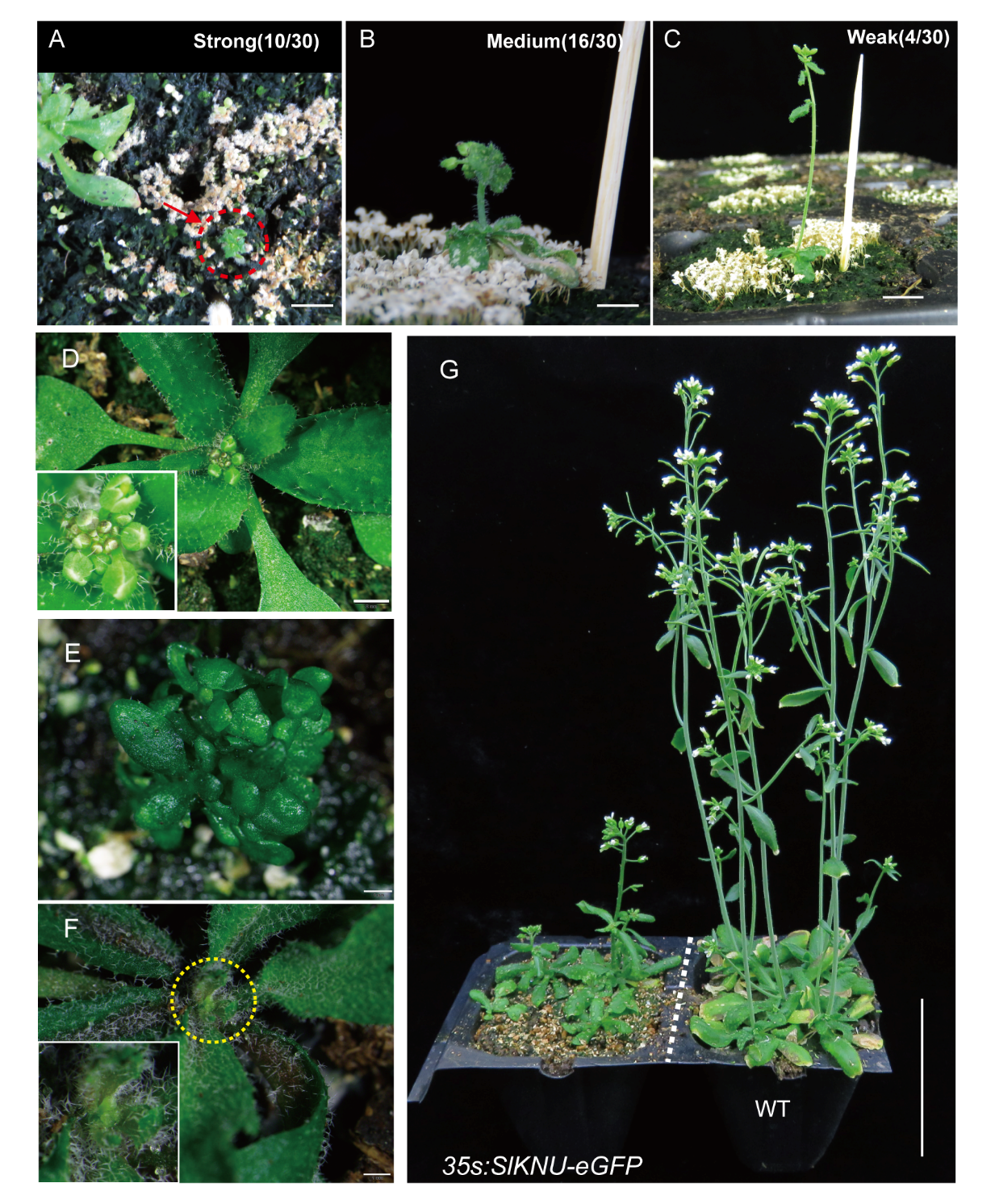


**Fig S8.** Phenotype of *35S:SlKNU-eGFP* transgenic plants. (**A-C**) Phenotypes of T1 generation of *35S:SlKNU-eGFP* transgenic plants. T1 line of a strong phenotype **(A)**, a moderate phenotype **(B)** and a weak phenotype (**C**), Scale bars= 3.5mm for **(A, B)**, and 5mm for **(C)**.**(D-F)** 3-week-old *Arabidopsis* seedlings. WT *Arabidopsis* **(D)**.*35S:SlKNU-eGFP* transgenic *Arabidopsis* showing loss of apical dominance (**E**) and reduced SAM activity (**F**). **(D-F),** Scale bars=2mm. (**G**) 5-week-old transgenic plants are shown on the left side, with WT plants on the right side. Scale bar=5cm.


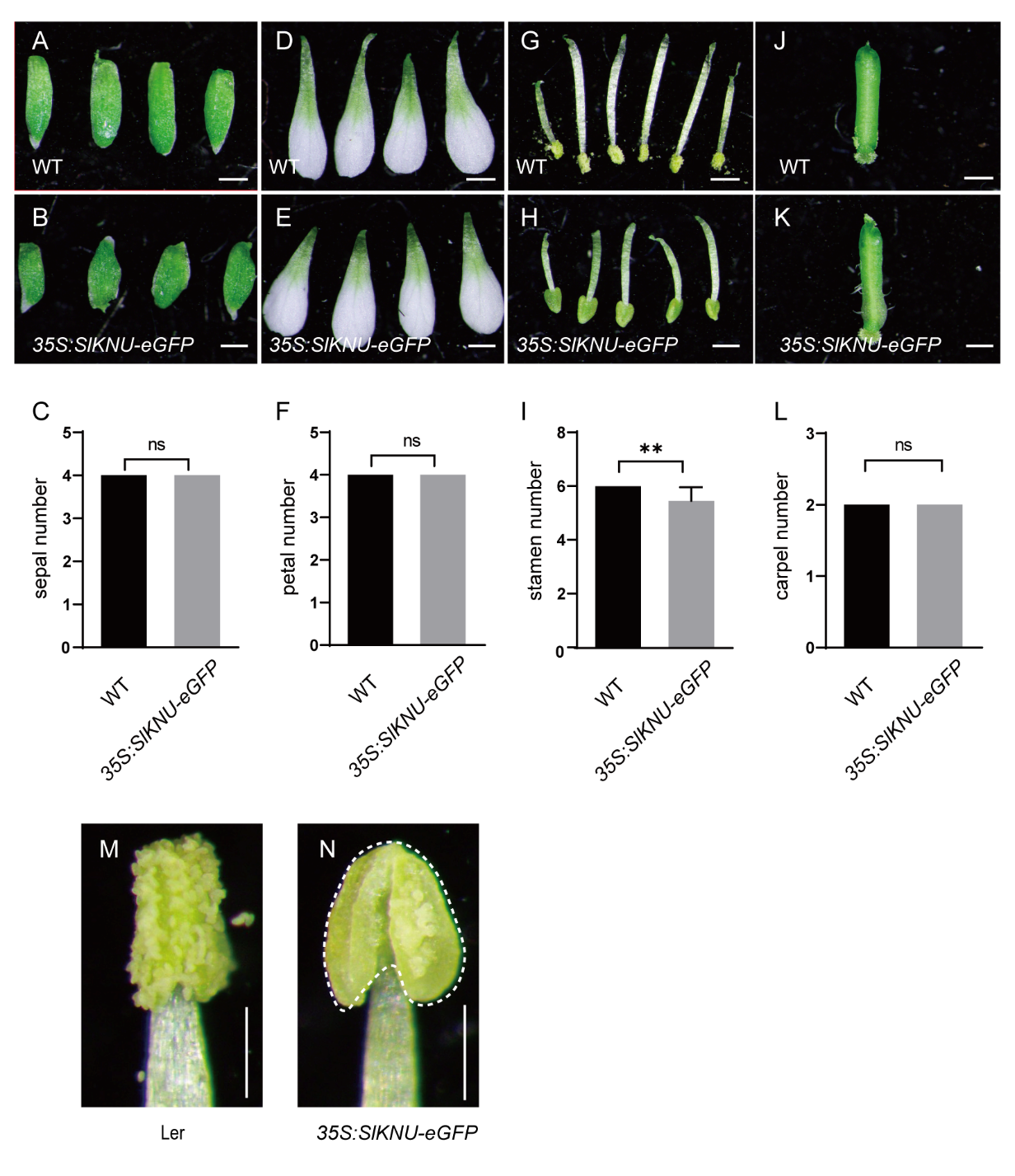


**Fig S9.** Comparison of floral organ numbers in WT and *35S:SlKNU-eGFP* transgenic Arabidopsis. **(A,B**) Sepals. WT (**A**), *35S:SlKNU-eGFP* (**B**). Sepal number analysis (**C**). (**D,E**) Petals. WT (**D**), *35S:SlKNU-eGFP* (**E**). Petal number data (**F**). (**G,H**) Stamens. WT (**G**), *35S:SlKNU-eGFP* **(H)**. (**I**) Stamen number display. (**J,K**) Carpels. WT (**J**), *35S:SlKNU-eGFP* (**K**). (**L**) Carpel number analysis. Scale bars = 500 μm. (***p*<0.01 Student’s t-test) (**M,N**) Stamen phenotype of WT (**M**) plants and *35S:SlKNU-eGFP* transgenic plants(**N**). Scale bars = 500 μm.


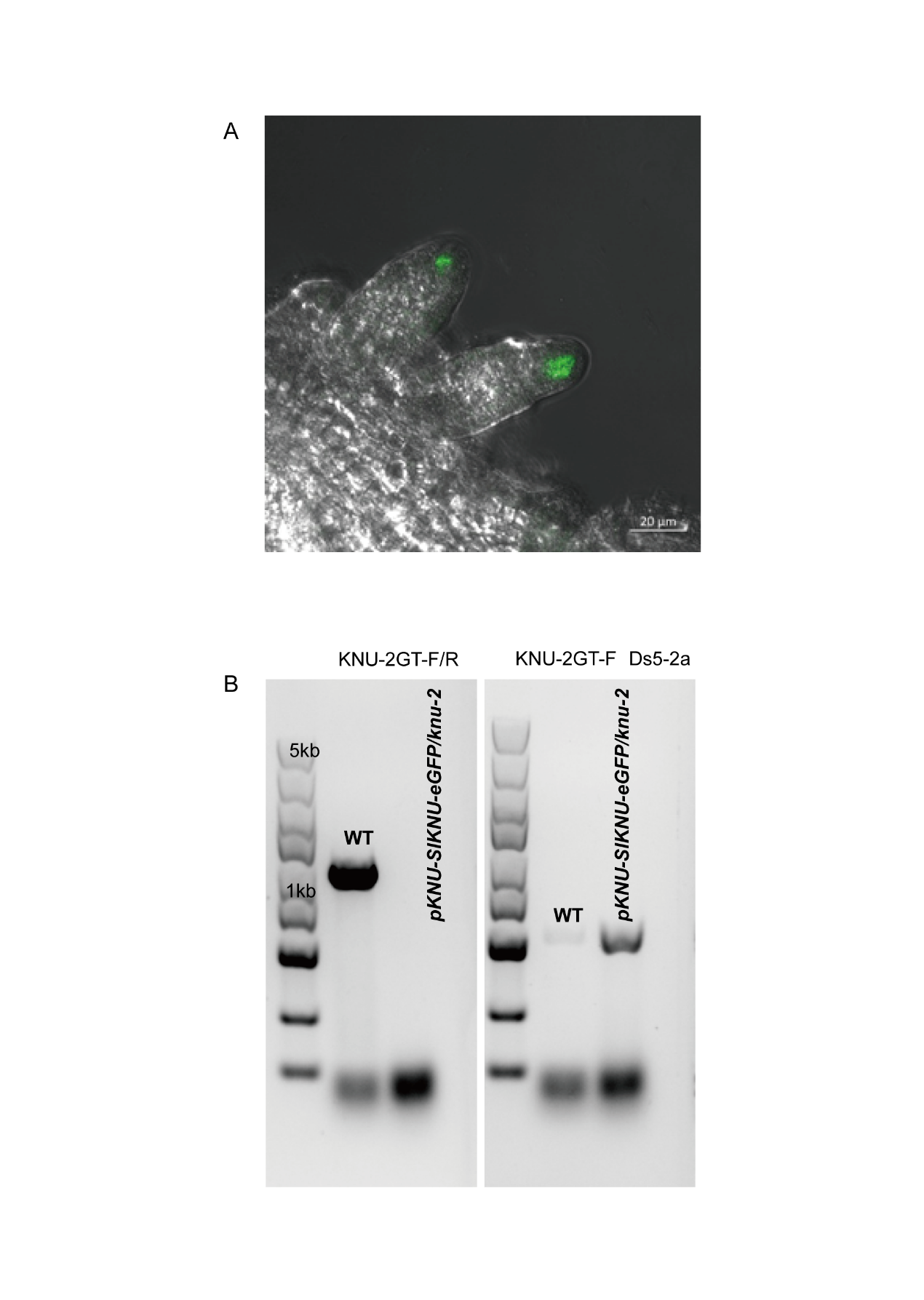


**Fig S10.** Identification of *knu-2 pKNU:SlKNU-eGFP*. Confocal observation revealed fluorescence signals of *pKNU:SlKNU-eGFP* in the megaspore mother cells.
