# Supplementary figures and images for: SlKNUCKLES regulates floral meristem activity and controls fruit size in Solanum lycopersicum
Source: Hortic Res. 2024 Nov 21;12(3):uhae331. doi: 10.1093/hr/uhae331 (PMC11879652; doi:10.1093/hr/uhae331)

A

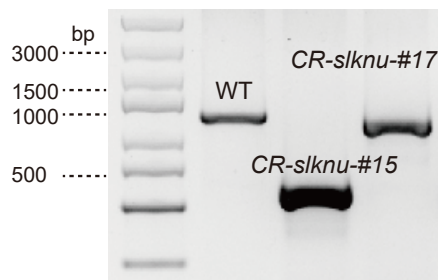

B

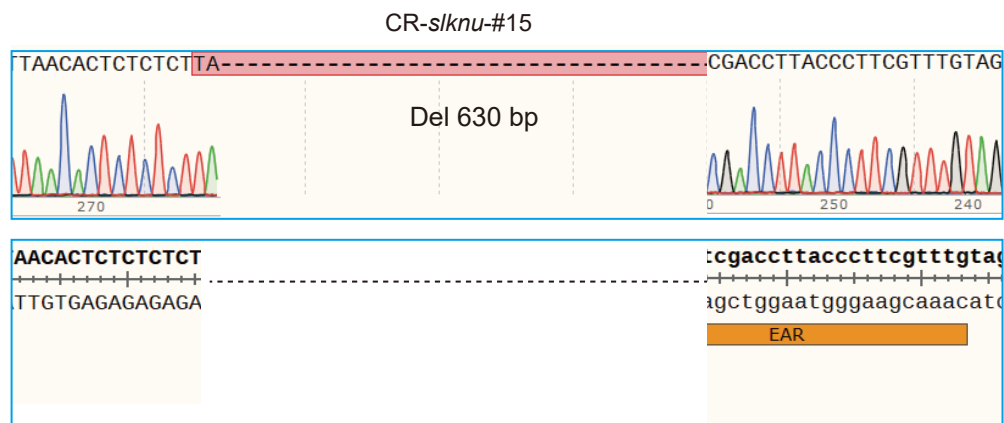

C

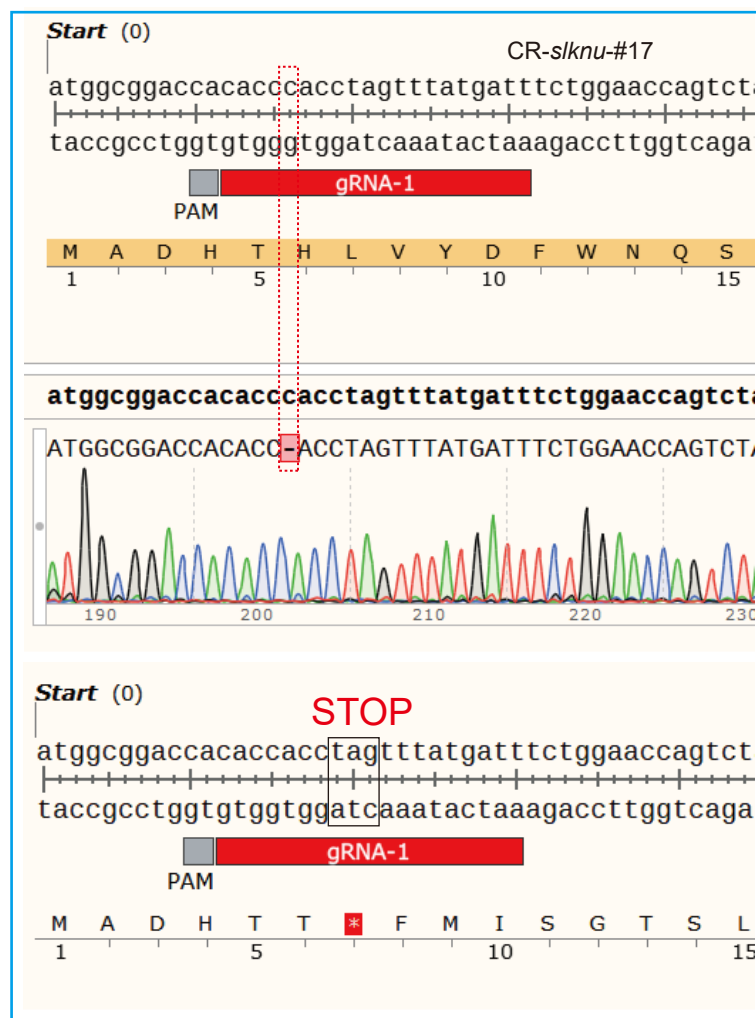

Supplement: Web_Material_uhae331 [file web_material_uhae331.zip › Fig. S1.pdf]

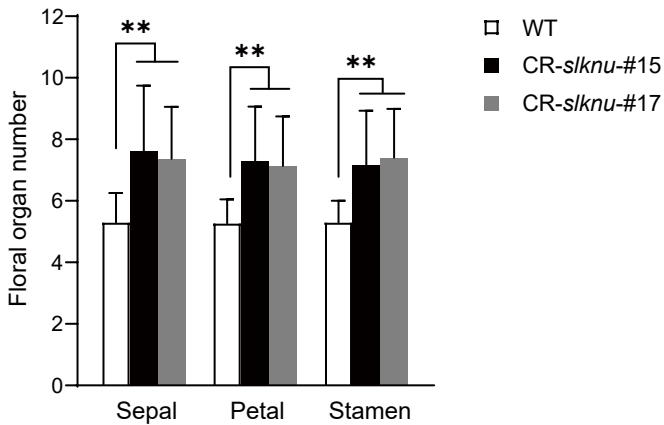

Supplement: Web_Material_uhae331 [file web_material_uhae331.zip › Fig. S2.pdf]

A

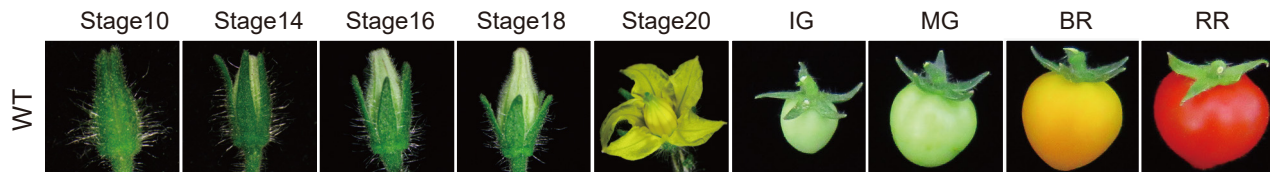

B

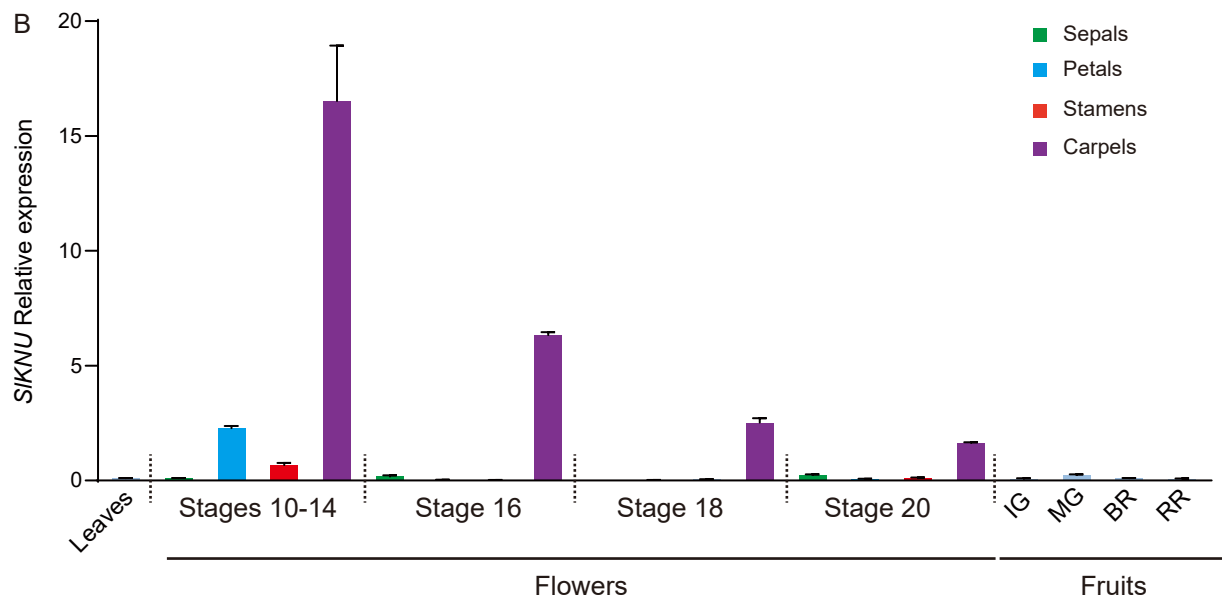

Supplement: Web_Material_uhae331 [file web_material_uhae331.zip › Fig. S3.pdf]

*35S:SIKNU-GR-3xmyc*

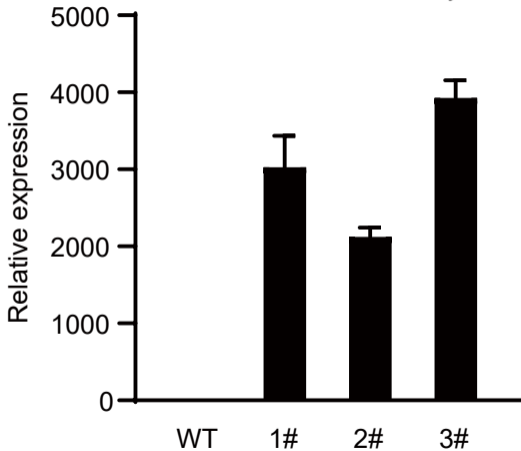

Supplement: Web_Material_uhae331 [file web_material_uhae331.zip › Fig. S4.pdf]

*Slc1v3*

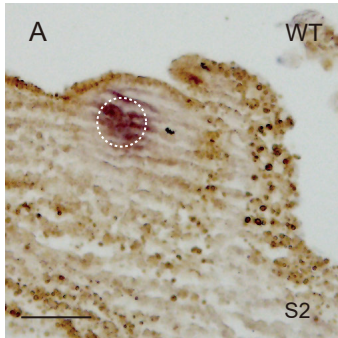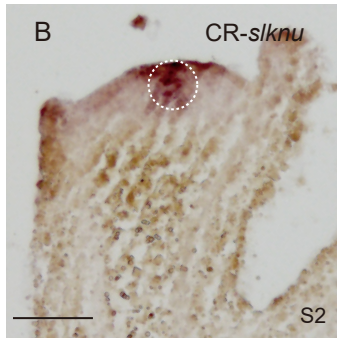

Supplement: Web_Material_uhae331 [file web_material_uhae331.zip › Fig. S5.pdf]

A

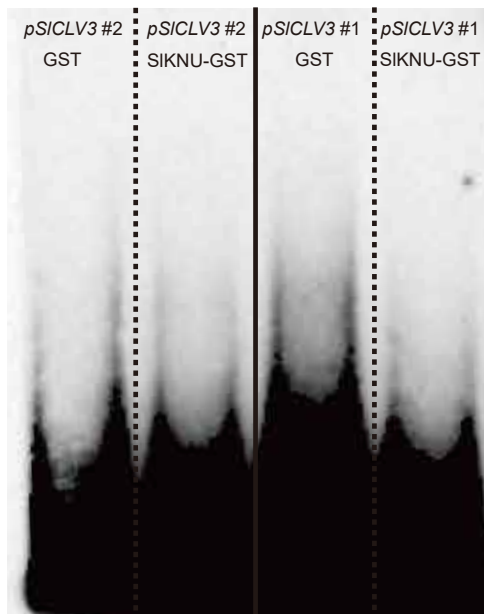

B

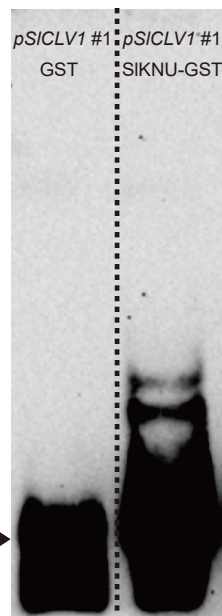

C

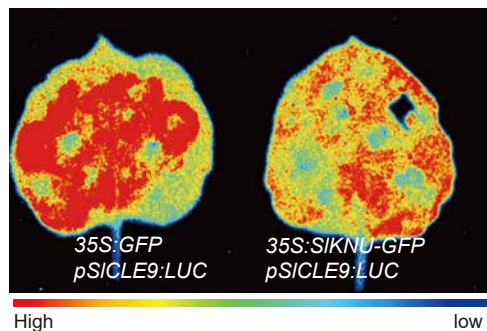

D

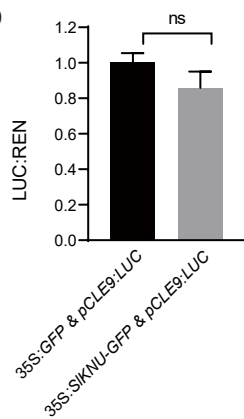

Supplement: Web_Material_uhae331 [file web_material_uhae331.zip › Fig. S6.pdf]

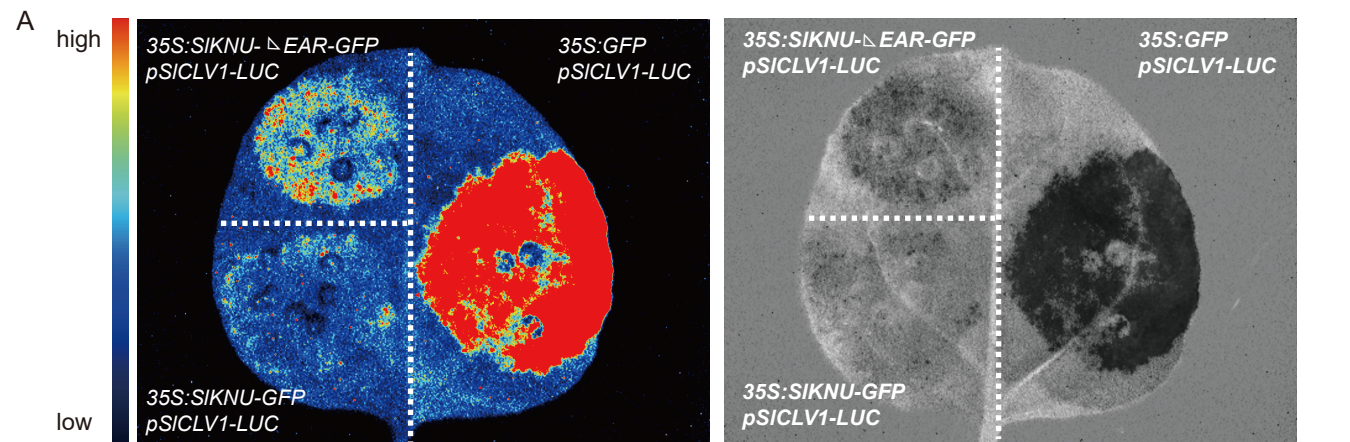

**B**

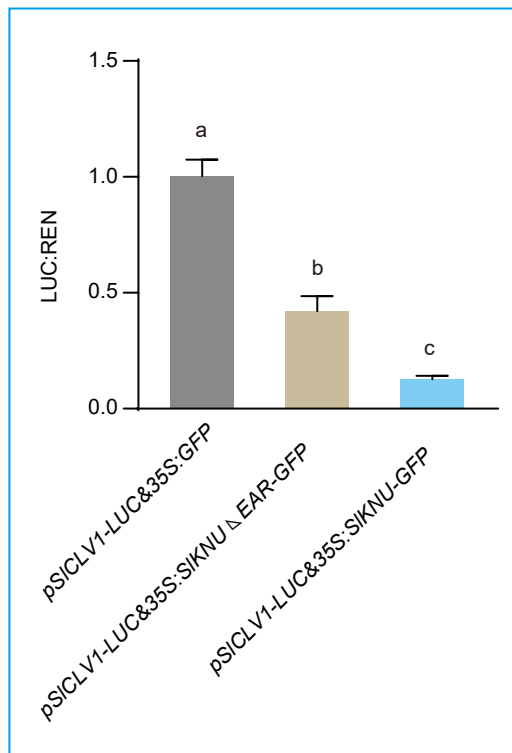

**C**

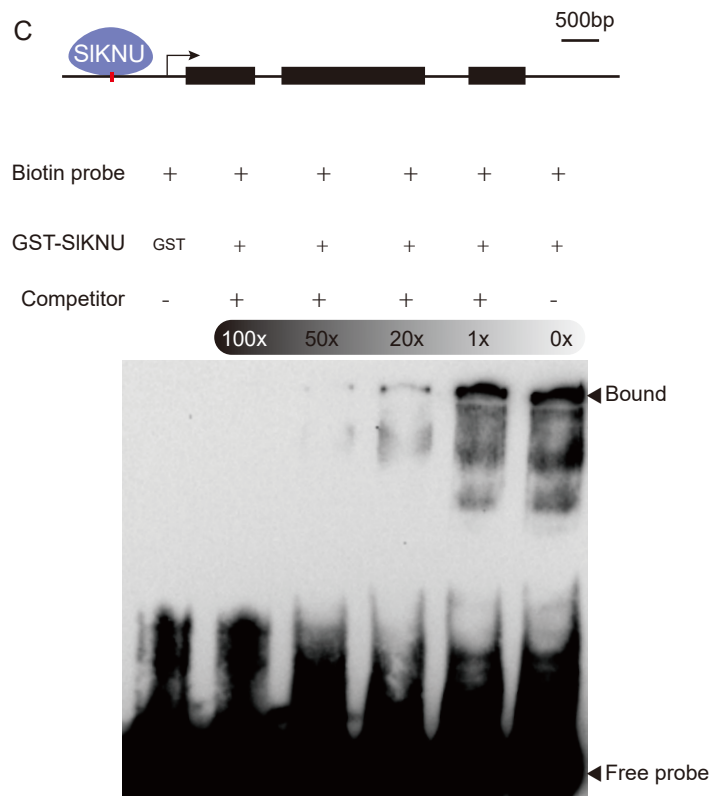

Supplement: Web_Material_uhae331 [file web_material_uhae331.zip › Fig. S7.pdf]

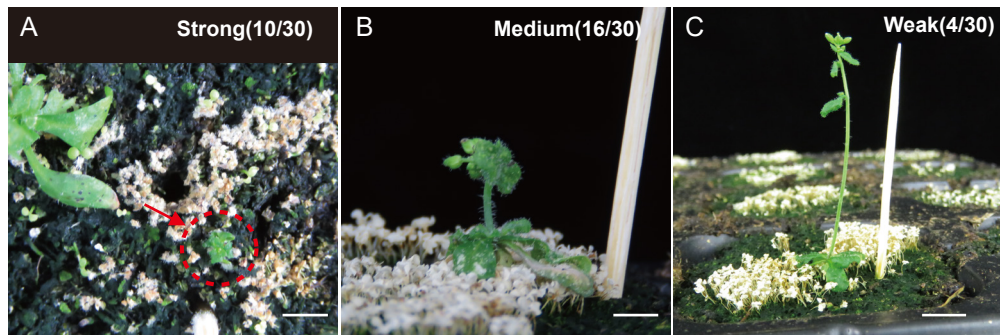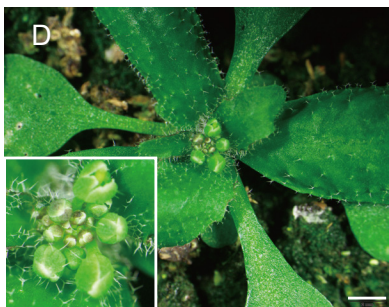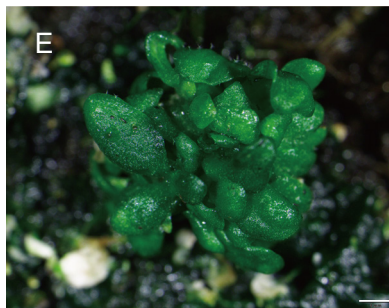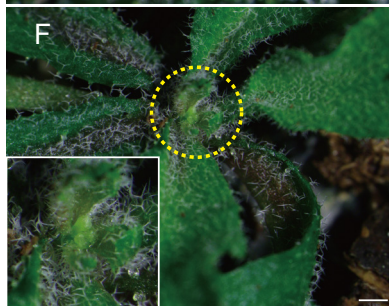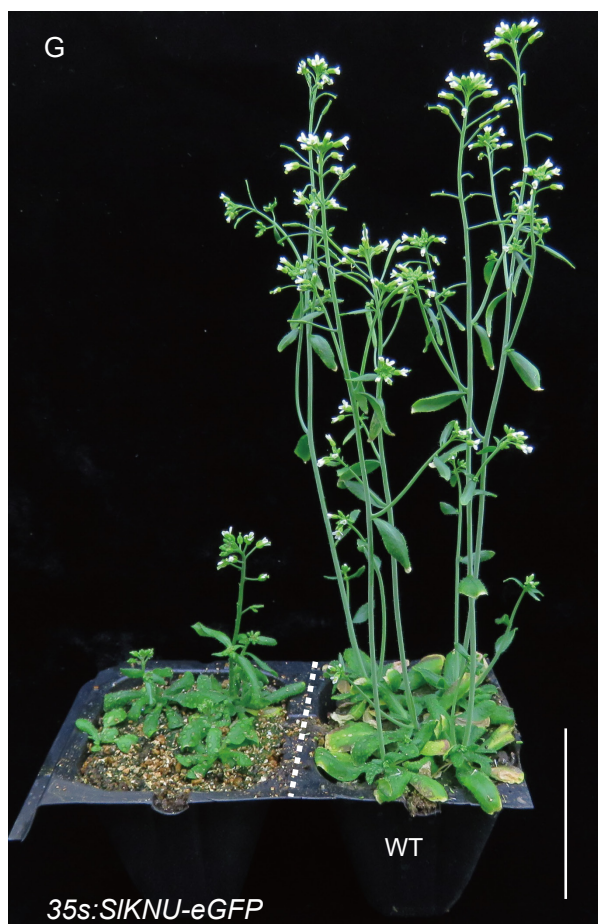

Supplement: Web_Material_uhae331 [file web_material_uhae331.zip › Fig. S8.pdf]

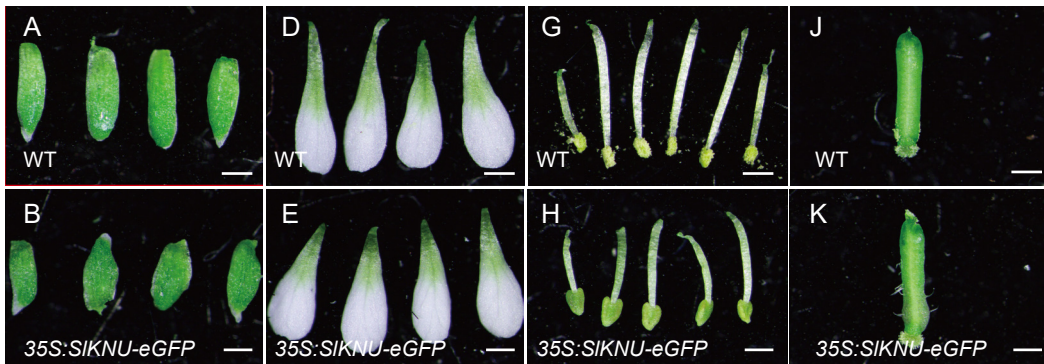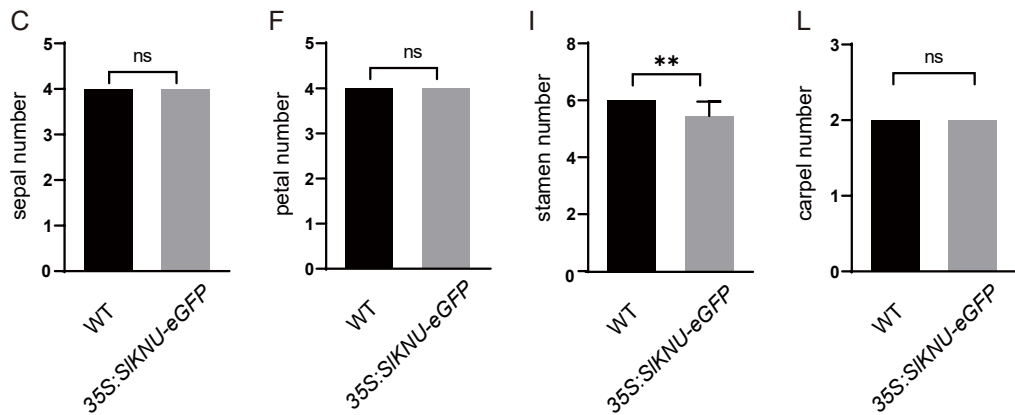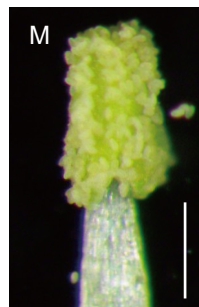

Ler

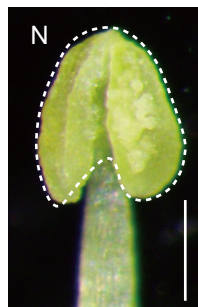

35S:SIKNU-eGFP

Supplement: Web_Material_uhae331 [file web_material_uhae331.zip › Fig. S9.pdf]

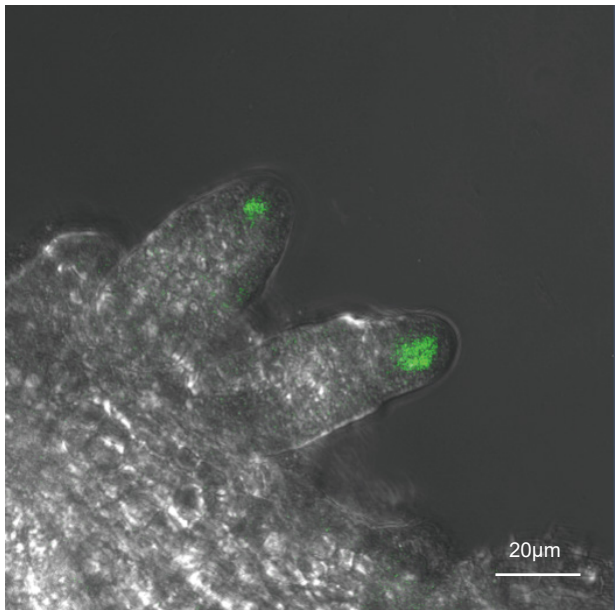

Supplement: Web_Material_uhae331 [file web_material_uhae331.zip › Fig. S10.pdf]
